# A computationally efficient physiologically comprehensive 3D–0D closed-loop model of the heart and circulation

**DOI:** 10.1016/j.cma.2021.114092

**Published:** 2021-08-18

**Authors:** Christoph M. Augustin, Matthias A.F. Gsell, Elias Karabelas, Erik Willemen, Frits W. Prinzen, Joost Lumens, Edward J. Vigmond, Gernot Plank

**Affiliations:** a Gottfried Schatz Research Center: Division of Biophysics, Medical University of Graz, Graz, Austria; b BioTechMed-Graz, Graz, Austria; c Department of Biomedical Engineering, CARIM School for Cardiovascular Diseases, Maastricht University, Maastricht, Netherlands; d IHU Liryc, Electrophysiology and Heart Modeling Institute, fondation Bordeaux Université, Pessac-Bordeaux, France

**Keywords:** Ventricular pressure–volume relation, Frank–Starling mechanism, Ventricular load

## Abstract

Computer models of cardiac electro-mechanics (EM) show promise as an effective means for the quantitative analysis of clinical data and, potentially, for predicting therapeutic responses. To realize such advanced applications methodological key challenges must be addressed. Enhanced computational efficiency and robustness is crucial to facilitate, within tractable time frames, model personalization, the simulation of prolonged observation periods under a broad range of conditions, and physiological completeness encompassing therapy-relevant mechanisms is needed to endow models with predictive capabilities beyond the mere replication of observations.

Here, we introduce a universal feature-complete cardiac EM modeling framework that builds on a flexible method for coupling a 3D model of bi-ventricular EM to the physiologically comprehensive 0D *CircAdapt* model representing atrial mechanics and closed-loop circulation. A detailed mathematical description is given and efficiency, robustness, and accuracy of numerical scheme and solver implementation are evaluated. After parameterization and stabilization of the coupled 3D–0D model to a limit cycle under baseline conditions, the model’s ability to replicate physiological behaviors is demonstrated, by simulating the transient response to alterations in loading conditions and contractility, as induced by experimental protocols used for assessing systolic and diastolic ventricular properties. Mechanistic completeness and computational efficiency of this novel model render advanced applications geared towards predicting acute outcomes of EM therapies feasible.

## Graphical abstract

**Graphic abstract:**
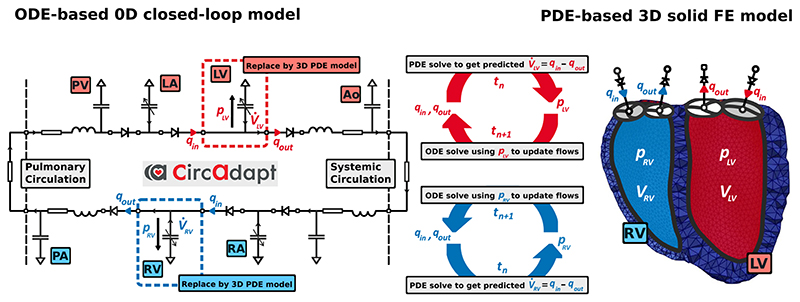

## 1 Introduction

Cardiovascular diseases (CVDs) are the primary cause of mortality and morbidity in industrialized nations, posing a significant burden on health care systems worldwide [1–3]. Despite continuous diagnostic and therapeutic advances, their optimal treatment remains a challenge [4]. In no small part, this is due to the complex multiphysics nature of cardiovascular function — the heart is an electrically controlled mechanical pump driving blood through the circulatory system. Advanced clinical modalities provide a wealth of disparate data, but effective tools allowing their comprehensive quantitative analysis are lacking. Computer models able to capture mechanistic relations between clinical observations quantitatively show promise to fill this void. In recent single physics cardiac electrophysiology (EP) studies, the added value of models in improving therapy stratification [5] and planning [6,7] has been demonstrated already.

Multiphysics models of cardiovascular EM are even more challenging to apply in a clinical context. Their utility depends on the ability to comprehensively represent mechanisms underlying a broader range of physiological function, and to tailor these to approximate – with acceptable fidelity – anatomy and cardiovascular function of a given patient. Such models are complex as all major mechanisms governing a heart beat bidirectionally interact with each other and, thus, must be taken into account. These comprise models of cardiac EP producing electrical activation and repolarization patterns that drive EM coupling to models of contractile function, cardiac mechanics describing deformation and stresses under given mechanical boundary and hemodynamic loading conditions imposed by the intra-thoracic embedding of the heart and the circulatory system, respectively.

Pumping function is regulated through a bidirectional interaction between the heart and both the systemic and pulmonary vascular systems. The circulatory system as an extracardiac factor imposes a pressure and volume load upon the heart and, *vice versa*, pressure and flow in the circulatory system are determined by the mechanical state of cardiac cavities. Optimal function depends on matching the coupling between these two systems [8]. From a physics point of view, coupling poses a fluid–structure interaction (FSI) problem, with pressure and blood flow velocity fields as coupling variables [9,10]. These are relevant for investigating flow patterns or wall shear stresses, but are less suitable for systems level investigations. Simpler, computationally less costly 0D and 1D lumped models have been preferred to provide appropriate hemodynamic loading conditions to the heart [11,12].

Most EM modeling studies consider ventricular afterload only represented by lumped 0D Windkessel type models comprising 2-, 3- or 4-elements [13–19], or, less common, by 1D models derived from Navier–Stokes equations [20–24]. The latter also account for pulse wave transmission and reflection, but identifying parameters is more challenging than for 0D models [25]. A fundamental limitation of isolated models of pre- and afterload is the lack of regulatory loops which respond to altered loading or contractility in one chamber by balancing preload conditions in all chambers until a new stable limit cycle with common compatible stroke volumes is reached. Isolated afterload models are thus best suited for approximating the immediate responses in a single beat [26], but less so for predicting transient behaviors over multiple beats. Closed-loop circulatory systems [27–31] take into account these feedback mechanisms and ascertain the conservation of blood volume throughout the cardiovascular system.

Achieving a flexible, robust, and efficient coupling of 3D EM models of cardiac chambers to a 0D closed-loop model of the circulatory system remains challenging. Hydrostatic pressure, *p*, in cavities and blood flow, *q*, between cavities and circulatory system serve as coupling variables that act as pressure boundary condition and impose volume constraints on the 3D cavity models. Previous studies addressed 3D solid–0D fluid coupling problems using simpler partitioned [32–34] or more advanced strongly coupled monolithic approaches [35–38,25,39]. Yet, reports on coupling of a closed-loop 0D to 3D solid models are sparse. Mostly simplified circuit models [37,33,38,40], were used for simulating a single heart beat, where fixed compliances and 0D chambers based on time-varying elastance models are used that do not account for pressure–volume relations or the Frank–Starling effect, respectively. Thus, attempts to demonstrate agreement with known physiological principles – fundamental to cardiac pump function – under experimental protocols requiring multibeat simulations have been limited.

Based on previous work on cardiac EM models [41–43] we report on the development of a monolithic 3D solid–0D fluid coupling approach. Feasibility is demonstrated by building a 3D canine bi-ventricular EM PDE model coupled to the state-of-the art *CircAdapt* model [27,44] – a non-linear 0D closed-loop ODE model of the cardiovascular system that implements dynamic adaptation processes based on physiological principles — to represent physiologically realistic atrial EM as well as systemic and pulmonary circulation. A detailed description of numerical underpinnings is given, including a complete mathematical description of the *CircAdapt* model in a single manuscript that has been lacking so far. Efficiency, robustness, and accuracy of numerical scheme and solver implementation are evaluated. The coupled model is first parameterized and stabilized to a limit cycle representing baseline conditions, and then rigorously tested by demonstrating its ability to predict physiological behaviors under experimental standard protocols altering loading conditions and contractility that are used for the experimental assessment of systolic and diastolic ventricular properties. Transient responses under these protocols are simulated over prolonged observation periods, covering up to 25 beats. The presented framework can be considered a first feature-complete realization of an universal cardiac EM simulator that can be applied, given appropriate parameterization and initialization, under a much broader range of protocols and conditions as any previously reported model.

## 2 Methodology

### 2.1 Experimental data acquisition

In a previous study, see [45], mongrel dog data were acquired to investigate the influence of different pacing protocols on cardiac mechanics, pump function and efficiency. The animals were handled according to the Dutch Law on Animal Experimentation (WOD) and the European Directive for the Protection of Vertebrate Animals Used for Experimental and Other Scientific Purposes (86/609/EU). The protocol was approved by the Maastricht University Experimental Animal Committee. Anatomical Magnetic Resonance Images (MRI) were acquired on a Philips Gyroscan 1.5 T (NT, Philips Medical Systems, Best, the Netherlands) using a standard synergy receiver coil for thorax examinations. Images of seven short-axis cross-sections of slice thickness 8mm with 0mm inter-slice distance were obtained to capture the whole heart. LV pressure and volume were determined using the conductance catheter technique (CD-Leycom, The Netherlands), see [46], and the signals were digitized at 1 kHz.

### 2.2 Biventricular finite element models

**Figure 1:**
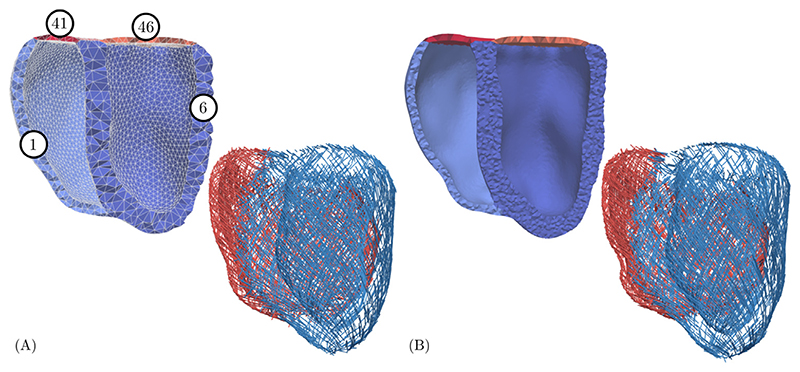
Coarse (A) and fine (B) resolution meshes with domain labels and corresponding fiber fields. Note the difference in fiber angles due to spatial resolution.

Multilabel segmentations of right ventricular (RV) (tag 36) and LV blood pool (tag 31) and of the LV myocardium (tag 1), see Fig. 1, were generated from seven MRI short axis slices using Seg3D [47]. Each slice was first segmented semi-automatically using thresholding techniques with manual correction. Segmentations were upsampled to isotropic resolution, followed by an automated iterative erosion and dilation smoothing scheme implemented in Meshtool [48]. The RV wall (tag 6) and lids representing the atrio-ventricular valves were automatically generated by dilation of the adjacent blood pool (tags 41 and 46). Biventricular multilabel meshes were created then from labeled segmentations [43] using the Computational Geometry Algorithms Library, CGAL (www.cgal.org) and subsequently smoothed with Meshtool [48]. A rule-based method according to [49] was applied to define fiber and sheet architecture, with fiber angles changing linearly from −60° at the epicardium to +60° at the endocardium [50]. Universal ventricular coordinates were computed [51] to support the flexible definition of stimulation sites and mechanical boundary conditions. Two meshes of different resolution were generated, a coarse mesh to reduce computational expenses and to facilitate the fast exploration of experimental protocols over prolonged observation periods, and a higher resolution mesh for investigating potential inaccuracies introduced by the coarser spatial resolution. For the coarse mesh, average edge lengths of ~ 3.4 mm and ~ 2.4 mm, were chosen in LV and RV, respectively, to ascertain that at least two elements were generated transmurally across the myocardial walls, as illustrated in Fig. 1. For the finer mesh, average edge lengths of ~ 1.3 mm and ~ 1.2 mm, were chosen in LV and RV, respectively.

### 2.3 Electromechanical PDE model

#### Tissue mechanics

Cardiac tissue is mechanically characterized as a hyperelastic, nearly incompressible, orthotropic material with a nonlinear stress–strain relationship. The deformation gradient **F** describes the deformation **u** of a body from the reference configuration *Ω*_0_(**X**) to the current configuration *Ω_t_*(**x**),

(1) $$F_{ij}=\frac{\partial x_{i}}{\partial X_{j}},\;i,j=1,2,3.$$

By convention, we denote *J* = det **F** > 0 and introduce the right Cauchy-Green tensor **C** = **F** ^⊤^ **F**. The nearly incompressible behavior is modeled by a multiplicative decomposition of the deformation gradient [52] of the form

(2) $$\text{F}=J^{1/3}\bar{\text{F}},\text{C}=J^{2/3}\bar{\text{C}},\;\text{with}\;\det\bar{\text{F}}=\det\bar{\text{C}}=1.$$

Mechanical deformation is described by Cauchy’s first equation of motion given as

(3) $$\rho_{0}ü(t,\text{X})-\text{Div}[\text{FS}(\text{u},\text{X})]=0\;\text{for}\;\text{X}\in \Omega_{0}\times (0,T),$$

with initial conditions

$$\text{u}(\text{X},0)=0,\;\dot{\text{u}}(\text{X},0)=0.$$

Here, *ρ*_0_ is the density in reference configuration; **ü** are nodal accelerations; $\dot{\text{u}}$ are nodal velocities; **S**(**u**, **X**) is the second Piola–Kirchhoff stress tensor; and Div denotes the divergence operator in the reference configuration.

The boundary of the bi-ventricular models was decomposed in three parts, $\partial \Omega_{0}={\bar{\Gamma }}_{\text{endo},0}\cup {\bar{\Gamma }}_{\text{epi},0}\cup {\bar{\Gamma }}_{\text{base},0}$, with ${\bar{\Gamma }}_{\text{endo},0}$ the endocardium, ${\bar{\Gamma }}_{\text{epi},0}$ the epicardium, and ${\bar{\Gamma }}_{\text{base},0}$ the base of the ventricles.

Normal stress boundary conditions were imposed on the endocardium

(4) $$\text{FS}(\text{u},\text{X}){\text{n}}_{0}^{\text{out}}(\text{X})=-p(t)J{\text{F}}^{-⊤}{\text{n}}_{0}^{\text{out}}(\text{X})\;\text{on}\;\Gamma_{\text{endo},0}\times (0,T)$$

with *p*(*t*) the pressure and ${\text{n}}_{0}^{\text{out}}$ the outer normal vector; omni-directional spring type boundary conditions constrained the ventricles at the basal cut plane ${\bar{\Gamma }}_{\text{base},0}$ [53]; and to simulate the mechanical constrains imposed by the pericardium spatially varying normal Robin boundary conditions were applied at the epicardium ${\bar{\Gamma }}_{\text{epi},0}$ [7].

Apart from external loads the deformation of cardiac tissue is in particular governed by active stresses intrinsically generated during contraction. To simulate both the active and passive properties of the tissue, the total stress **S** is additively decomposed according to

(5) $$\text{S}={\text{S}}_{\text{p}}+{\text{S}}_{\text{a}},$$

where **S**_p_ and **S**_a_ refer to the passive and active stresses, respectively.

#### Passive stress

Passive stresses are modeled based on the constitutive equation

(6) $${\text{S}}_{p}=2\frac{\partial \Psi (\text{C})}{\partial \text{C}},$$

where *Ψ* is a strain–energy function to model the orthotropic behavior of cardiac tissue. The prevailing orientation of myocytes, referred to as fiber orientation, is denoted as **f**_0_. Individual myocytes are surrounded and interconnected by collagen, forming sheets, which is described by the sheet orientation **s**_0_, perpendicular to **f**_0_. Together with the sheet-normal axis **n**_0_, orthogonal to the sheet and the fiber orientations, this forms a right-handed orthonormal set of basis vectors.

Following Usyk et al. [54] the orthotropic constitutive relation is defined as

(7) $$\Psi (\text{C})=\frac{\kappa }{2}(\log J{)}^{2}+\frac{a}{2}[\exp(Q)-1],$$

where the first term is the volumetric energy with the bulk modulus *κ* ≫ 0 kPa which penalizes local volume changes to enforce near incompressible behavior of the tissue, parameter *a* is a stress-like scaling parameter, and the term in the exponent is

(8) $$Q=b_{\text{ff}}{\bar{E}}_{\text{ff}}^{2}+b_{\text{ss}}{\bar{E}}_{\text{ss}}^{2}+b_{\text{nn}}{\bar{E}}_{\text{nn}}^{2}+b_{\text{fs}}\left({\bar{E}}_{\text{fs}}^{2}+{\bar{E}}_{\text{sf}}^{2}\right)+b_{\text{fn}}\left({\bar{E}}_{\text{fn}}^{2}+{\bar{E}}_{\text{nf}}^{2}\right)+b_{\text{ns}}\left({\bar{E}}_{\text{ns}}^{2}+{\bar{E}}_{\text{sn}}^{2}\right).$$

Here, *b*_•_ are dimensionless parameters and the directional strains read

$${\bar{E}}_{\text{ff}}={\text{f}}_{0}\cdot \bar{\text{E}}{\text{f}}_{0},{\bar{E}}_{\text{ss}}={\text{s}}_{0}\cdot \bar{\text{E}}{\text{s}}_{0},{\bar{E}}_{\text{nn}}={\text{n}}_{0}\cdot \bar{\text{E}}{\text{n}}_{0},{\bar{E}}_{\text{fs}}={\text{f}}_{0}\cdot \bar{\text{E}}{\text{s}}_{0},{\bar{E}}_{\text{fn}}={\text{f}}_{0}\cdot \bar{\text{E}}{\text{n}}_{0},$$

$${\bar{E}}_{\text{ns}}={\text{n}}_{0}\cdot \bar{\text{E}}{\text{s}}_{0},{\bar{E}}_{\text{sf}}={\text{s}}_{0}\cdot \bar{\text{E}}{\text{f}}_{0},{\bar{E}}_{\text{nf}}={\text{n}}_{0}\cdot \bar{\text{E}}{\text{f}}_{0},{\bar{E}}_{\text{sn}}={\text{s}}_{0}\cdot \bar{\text{E}}{\text{n}}_{0},$$

with $\bar{\text{E}}=\frac{1}{2}(\bar{\text{C}}-\text{I})$ the modified isochoric Green–Lagrange strain tensor. All passive material parameters are given in Table 2.

#### Active stress

Stresses due to active contraction are assumed to be orthotropic with full contractile force along the myocyte fiber orientation **f**_0_ and 40% contractile force along the sheet orientation **s**_0_ [55,56]. Thus, the active stress tensor is defined as

(9) $${\text{S}}_{\text{a}}=S_{\text{a}}{\left({\text{f}}_{0}\cdot \text{C}{\text{f}}_{0}\right)}^{-1}{\text{f}}_{0}⊗{\text{f}}_{0}+0.4\;S_{\text{a}}{\left({\text{s}}_{0}\cdot \text{C}{\text{s}}_{0}\right)}^{-1}{\text{s}}_{0}⊗{\text{s}}_{0},$$

where *S_a_* is the scalar active stress describing the contractile force. A simplified phenomenological contractile model was used to represent active stress generation [26]. Owing to its small number of parameters and its direct relation to clinically measurable quantities such as peak pressure, and the maximum rate of rise of pressure this model is fairly easy to fit and thus very suitable for being used in clinical EM modeling studies. Briefly, the active stress transient is given by

(10) $$S_{\text{a}}(t,\lambda )=S_{\text{peak}}\phi (\lambda )\tanh^{2}\left(\frac{t_{\text{s}}}{\tau_{\text{c}}}\right)\tanh^{2}\left(\frac{t_{\text{dur}}-t_{\text{s}}}{\tau_{\text{r}}}\right),\;\text{for}\;0<t_{\text{s}}<t_{\text{dur}},$$

with

(11) $$\phi =\tanh\left(\text{ld}\left(\lambda -\lambda_{0}\right)\right),\;\tau_{\text{c}}=\tau_{{\text{c}}_{0}}+{\text{ld}}_{\text{up}}(1-\phi ),\;t_{\text{s}}=t-t_{\text{a}}-t_{\text{emd}}$$

and *t*_s_ is the onset of contraction; *ϕ*(*λ*) is a non-linear length-dependent function in which *λ* is the fiber stretch and *λ*_0_ is the lower limit of fiber stretch below where no further active tension is generated; *t*_a_ is the local activation time from (Eq. 12), defined when the local transmembrane potential passes the threshold voltage *V*_m,thresh_; *t*_emd_ is the EM delay between the onsets of electrical depolarization and active stress generation; *S*_peak_ is the peak isometric tension; *t*_dur_ is the duration of active stress transient; *τ*_c_ is time constant of contraction; *τ*_c_0__ is the baseline time constant of contraction; ld_up_ is the length-dependence of *τ*_c_; *τ*_r_ is the time constant of relaxation; and ld is the degree of length dependence. For the parameter values used in the simulations see Table 2. Note that active stresses in this simplified model are only length-dependent, but dependence on fiber velocity, $\dot{\lambda }$, is ignored.

#### Electrophysiology

A recently developed reaction-eikonal (R-E) model [57] was employed to generate electrical activation sequences which serve as a trigger for active stress generation in cardiac tissue. The hybrid R-E model combines a standard reaction–diffusion (R–D) model based on the monodomain equation with an eikonal model. Briefly, the eikonal equation is given as

(12) $$\{\begin{array}{llll} \sqrt{\nabla_{\text{X}}t_{\text{a}}^{⊤}\text{V}\nabla_{\text{X}}t_{\text{a}}} & = & 1 & \text{in}\;\Omega_{0},\\ t_{\text{a}} & = & t_{0} & \text{on}\;\Gamma_{0}^{*}, \end{array}$$

where (∇_X_) is the gradient with respect to the end-diastolic reference configuration *Ω*_0_; *t*_a_ is a positive function describing the wavefront arrival time at location **X** ∈ *Ω*_0_; and *t*_0_ are initial activations at locations $\Gamma_{0}^{*}\subseteq \Gamma_{\text{N},0}$. The symmetric positive definite 3 × 3 tensor **V**(**X**) holds the squared velocities (*v*_f_(**X**), *v*_s_(**X**), *v*_n_(**X**)) associated to the tissue’s eigenaxes **f**_0_, **s**_0_, and **n**_0_. The arrival time function *t*_a_(**X**) was subsequently used in a modified monodomain R–D model given as

(13) $$\beta C_{\text{m}}\frac{\partial V_{\text{m}}}{\partial t}=\nabla_{\text{X}}\cdot \sigma_{\text{m}}\nabla_{\text{X}}V_{\text{m}}-\beta I_{\text{ion}}+I_{\text{foot}},$$

with *β* the membrane surface-to-volume ratio; *C*_m_ the membrane capacitance; *V*_m_ the unknown transmembrane voltage; *σ*_m_ the monodomain conductivity tensor which holds the scalar conductivities (*g*_f_(*X*), *g*_s_(*X*), *g*_n_(*X*)) and is coupled to **V**(**X**) proportionally [58]; and *I*_ion_ the membrane ionic current density. Additionally, an arrival time dependent foot current, *I*_foot_(*t*_a_), was added which is designed to mimic subthreshold electrotonic currents to produce a physiological foot of the action potential. The key advantage of the R-E model is its ability to compute activation sequences at coarser spatial resolutions that are not afflicted by the spatial undersampling artifacts leading to conduction slowing or even numerical conduction block, as it is observed in standard R–D models [59]. Ventricular EP was represented by the ten Tusscher–Noble–Noble–Panfilov model of the human ventricular myocyte [60].

#### Computation of volumes

To compute the flow across the interface between 3D cavities and the 0D cardiovascular system, the cavitary volume of each chamber that is described as a 3D PDE model has to be tracked as a function of time: *V* ^PDE^(**x**, *t*). A reduction in cavitary volume

$$\frac{\partial V^{\text{PDE}}(\text{x},t)}{\partial t}<0,$$

drives a positive flow into the circulatory system. In a pure EM simulation context where the fluid domain is not modeled explicitly, the cavitary blood pool volume is not discretized, only the surface *Γ* enclosing the volume is known. Assuming that the entire surface of the cavitary volume is available, that is, also the faces representing the valves are explicitly discretized, the enclosed volume *V* ^PDE^ can be computed from this surface using the divergence theorem

(14) $$V^{\text{PDE}}(\text{u},t)=V^{\text{PDE}}(\text{x},t)=\frac{1}{3}\int_{\Gamma_{t}}\text{x}\cdot \text{n}\;\text{d}\Gamma_{t}.$$

Using this approach, the volume *V* ^PDE^(**x**, *t*) can be computed for each state of deformation at time *t* and the flow can be derived by a numerical approximation using a difference quotient.

### 2.4 *Lumped ODE model of the circulatory system*: *the* CircAdapt *model*

**Figure 2:**
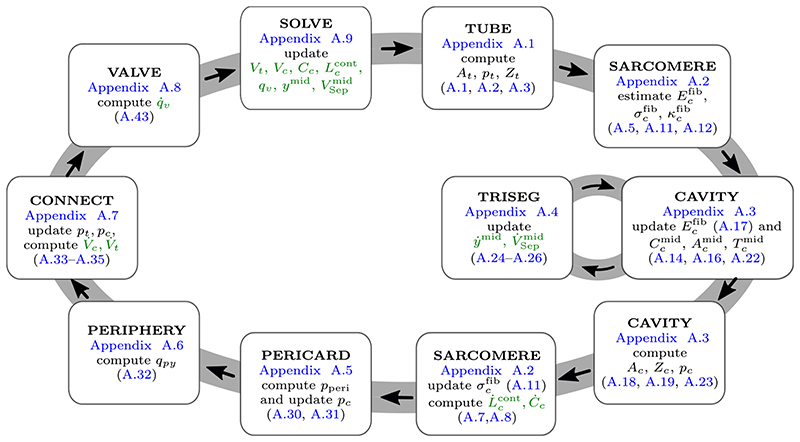
Solution process of the lumped ODE model of the circulatory system. The *CircAdapt* model connects tubes (*t*), cavities (*c*), valves (*v*), and pulmonary and systemic periphery (*py*). In each timestep the ODE system is solved using a Runge-Kutta-Fehlberg method, see Appendix A.9, to update the ODE variables (in green), i.e., volumes of tubes (*V_t_*) and cavities (*V_c_*); sarcomere contractility (*C_c_*) and sarcomere length $\left(L_{c}^{\text{cont}}\right)$ for each of the cavities and the septum; flow over valves (*q_v_*); and septal midwall volume $\left(V_{\text{Sep}}^{\text{mid}}\right)$ and radius (*y* ^mid^), see Fig. A.9c. In the following steps the updated variables are used to compute current pressures (*p_c_*, *p_t_*), cross sectional areas (*A_c_*, *A_t_*), and impedances (*Z_c_*, *Z_t_*) for tubes and cavities; fiber strain $\left(E_{c}^{\text{fib}}\right)$, fiber stiffness $\left(\kappa_{c}^{\text{fib}}\right)$, and fiber stress $\left(\sigma_{c}^{\text{fib}}\right)$ for the sarcomeres of each cavity and the septum; midwall curvature $\left(C_{c}^{\text{mid}}\right)$, midwall area $\left(A_{c}^{\text{mid}}\right)$, and midwall tension $\left(T_{c}^{\text{mid}}\right)$ for each cavity and the septum; pericardial pressure *p*_peri_; and flow over the systemic and pulmonary periphery *q_py_*.. (For interpretation of the references to color in this figure legend, the reader is referred to the web version of this article.) *Source:* Based on [44].

*CircAdapt* [27], as shown schematically in Fig. 2, is a lumped 0D model of heart and circulation. It enables real-time simulation of cardiovascular system dynamics under a wide variety of physiological and pathophysiological situations. The entire cardiovascular system is modeled as a concatenation of modules: a tube module representing the systemic and pulmonary arteries and veins (Appendix A.1); a chamber module modeling actively contracting chambers, i.e., left and right atria and ventricles (Appendix A.3), respectively, where myofiber mechanics and contraction is described by a sarcomere module (Appendix A.2); following Lumens et al. [61] this also includes inter-ventricular mechanical interaction through the inter-ventricular septum (Appendix A.4); a valve module representing the aortic, mitral, pulmonary, and tricuspid valves (Appendix A.8); a module representing systemic and pulmonary peripheral microvasculatures (Appendix A.6); and a module accounting for effects of the pericardium (Appendix A.5). The modules are connected by flows over valves and venous-atrial inlets (Appendix A.7). The whole lumped model consists of 26 ordinary differential equations (ODEs) which are solved using an adaptive Runge–Kutta–Fehlberg method (RKF45) (Appendix A.9).

In Appendix A the mathematical underpinnings of the *CircAdapt* model are outlined. Briefly, cavity pressures and cavity volumes are interconnected as follows: volumes regulate cavity wall areas, which in turn determine strain of the myofibers in the wall. Strain is used to calculate myofiber stress, (A.9), (A.10), which drives wall tension in each cardiac wall (A.22). Using Laplace’s law, transmural pressure is calculated from wall tension and curvature for each wall (A.23). Cavity pressures are found by adding the transmural pressures to the intra-pericardial pressure surrounding the myocardial walls (A.31). Consecutively, cavity pressures are used to update flow over valves (A.43) and thus intra-cavitary volumes (A.34).

A significant advantage of the modular setup of the model is that a simple 0D module can be straightforwardly replaced by the more complicated finite element (FE) model in Section 2.2. In this setup *CircAdapt* provides realistic boundary conditions to the FE problem, see Section 2.5.

The version of the *CircAdapt* model used for all simulations has been published previously [44] and can also be downloaded from the *CircAdapt* website (http://www.circadapt.org).

### 2.5 PDE-ODE coupling

We introduce the set of cavities 𝒞 = {LV, RV, LA, RA}, with the left ventricle (LV), the left atrium (LA), the right ventricle (RV), and the right atrium (RA); the set of cavities $C^{\text{PDE}}\subseteq C$ that are modeled as a 3D PDE model; and the set of cavities $C^{\text{ODE}}=C\setminus C^{\text{PDE}}$ that are modeled as a 0D ODE model. Coupling between PDE and ODE models can be achieved in various ways. Fundamentally, the problem is to find the new state of deformation **u**_*n*+1_ as a function of the pressure *p*_*n*+1_ in a given cavity at time *n* + 1. The pressure *p*_*n*+1_ is applied as a Neumann boundary condition at the cavitary surface, see Eq. (4). This pressure is not known and has to be determined in a way which depends on the current state of the cavity. Basically, two scenarios have to be considered: (i) when all valves are closed, the cavity is in an isovolumetric state. That is, the muscle enclosing the cavity may deform, but the volume has to remain constant. Therefore if active stresses vary over time during an isovolumetric phase, the pressure *p*_*n*+1_ in the cavity has to vary as well to keep the cavitary volume constant; (ii) when at least one valve is open or regurging, the cavitary volume is changing. In this case the pressure *p*_*n*+1_ is influenced by the state of the circulatory system or of a connected cavity. Thus *p*_*n*+1_ has to be determined in a way that matches mechanical deformation and state of the system. Pressure *p*_*n*+1_ in the cardiovascular system depends on flow and flow rate which are governed by cardiac deformation and as such the two models are tightly bidirectionally coupled.

The simplest approach for the PDE–ODE coupling is to determine *p*_*n*+1_ using a partitioned scheme [32–34]. During ejection phases this is achieved by updating cavity volumes and flow based on the current prediction on the change in the state of deformation under the currently predicted pressure *p*_*n*+1_. In this scenario the pressure boundary condition in each non-linear solver step is modified within each Newton iteration *k*. The new prediction $p_{n+1}^{k+1}$ is then prescribed explicitly as a Neumann boundary condition. While this partitioned approach is easy to implement and may be incorporated into an existing FE solver package without difficulty, it may introduce inaccuracies during ejection phases and its convergence may deteriorate during isovolumetric phases [38]. Instabilities are related to the so-called balloon dilemma [62] and stem from the problem of estimating the change in pressure necessary to maintain the volume. Inherently, this requires to know the pressure–volume (*pV*) relation of the cavity at this given point in time. However, this knowledge on chamber elastance is not available and thus iterative estimates are necessary to gradually inflate or deflate a cavity to its prescribed volume. As the elastance properties of the cavities are highly non-linear, an overestimation may induce oscillations and an underestimation may lead to very slow convergence and a punitively large numbers of Newton iterations.

A more elaborate approach is to treat *p*_*n*+1_ as an additional unknown in a monolithic scheme [33,63,38,64]. In addition to the equilibrium equations (3)–(4) this requires one further equation for each cavity *c* ∈ 𝒞^PDE^. Using this approach, we get $N_{\text{cav}}=\left|C^{\text{PDE}}\right|$, the number of PDE cavities, additional equations of the form

(15) $$V_{c}^{\text{PDE}}(\text{u},t)-V_{c}^{\text{ODE}}\left(p_{c},t\right)=0,\;c\in C^{\text{PDE}}$$

where *V_c_* ^PDE^(**u**, *t*) is the cavity volume computed as the integral over the current surface *Γ_c,t_*, see Eq. (14), and *V_c_* ^ODE^(*p_c_*, *t*) is the cavity volumes as predicted by the *CircAdapt* model for the intra-cavitary pressure *p_c_*, see Section 2.4 and Appendix A.

We write ${\underline{p}}_{C}={\left[p_{c}\right]}_{c\in C}$ for the vector of up to 1 ≤ *N_cav_* ≤ 4 pressure unknowns. Then, linearization of the variational problem, see Appendix B.2, a Galerkin FE discretization, see Appendix B.3, and a time integration using a generalized-α scheme, see Appendix C, result in solving the block system to find $\delta \underline{u}\in {\mathbb{R}}^{3N}$ and $\delta {\underline{p}}_{C}\in {\mathbb{R}}^{N_{\text{cav}}}$ such that

(16) $${\text{K}}^{\prime }\left({\underline{u}}^{k},{\underline{p}}_{C}^{k}\right)\left(\begin{array}{c} \delta \underline{u}\\ \delta {\underline{p}}_{C} \end{array}\right)=-\underline{K}\left({\underline{u}}^{k},{\underline{p}}_{C}^{k}\right),\;\underline{K}\left({\underline{u}}^{k},{\underline{p}}_{C}^{k}\right):=\left(\begin{array}{l} {\underline{R}}_{\alpha }\left({\underline{u}}^{k},{\underline{p}}_{C}^{k}\right)\\ {\underline{R}}_{\text{p}}\left({\underline{u}}^{k},{\underline{p}}_{C}^{k}\right) \end{array}\right),$$

with the updates

(17) $${\underline{u}}^{k+1}={\underline{u}}^{k}+\delta \underline{u},$$

(18) $${\underline{p}}_{C}^{k+1}={\underline{p}}_{C}^{k}+\delta {\underline{p}}_{C}.$$

Here, ${\underline{u}}^{k}\in {\mathbb{R}}^{3N}$ and ${\underline{p}}_{C}^{k}\in {\mathbb{R}}^{N_{\text{cav}}}$ are the solution vectors at the *k*th Newton step. The block tangent stiffness matrix **K**′ is assembled according to Eqs. (B.20)-(B.24) and (C.12) and the right hand side vector ${\underline{R}}_{\alpha }$ according to Eqs. (B.25) and (B.26) and (C.9). The residual ${\underline{R}}_{\text{p}}$ which measures the accuracy of the current coupling is the discrete version of (15), i.e.,

(19) $${\underline{R}}_{\text{p}}\underline{(u^{k}},{\underline{p}}_{C}^{k}):={\underline{V}}^{\text{PDE}}\left({\underline{u}}^{k}\right)-{\underline{V}}^{\text{ODE}}\left({\underline{p}}_{C}^{k}\right).$$

**Figure 3:**
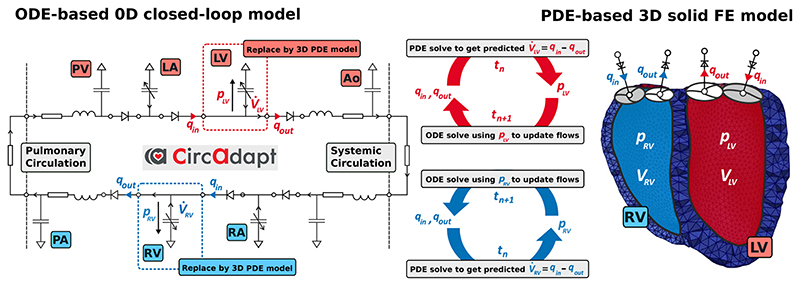
Schematic showing the coupling of the 0D ODE model, represented by the electrical equivalent circuit, to the 3D PDE model, represented by the FE mesh. In this case the ventricles (LV, RV) in the lumped model are replaced by 3D PDEs, while the atria (LA, RA) are modeled as lumped cavities in the *CircAdapt* model. Volume changes of the 3D cavities ${\dot{V}}_{\text{LV}}$, ${\dot{V}}_{\text{RV}}$ are driven by flow *q*_•_ of blood over valves and outlets computed by the 0D model. In turn, updated pressures *p*_LV_ and *p*_RV_ are used as an input to the lumped model in the next time step *t*_n+1_. The opening and closure of valves is only modeled in the lumped model and in the 3D model triangulated membranes are used to close the LV and RV cavities. Red colors indicate oxygenated and blue colors de-oxygenated blood. (For interpretation of the references to color in this figure legend, the reader is referred to the web version of this article.)

The whole procedure to perform the PDE–ODE coupling is given in Algorithm 1. In short, volume changes of the 3D cavities are driven by flow of blood over valves and outlets computed by the 0D model. In turn, updated pressures in the 3D cavities are used as an input to the lumped model in the next time step. Note that Eq. (16) is a block system with $\delta {\underline{p}}_{C}$ holding at most four unknowns. Hence, we can apply a Schur complement approach for a small number of constraints, as described in Appendix D, to simplify the numerical solution of this linearized system, see Section 2.8. the cavitary volume of each chamber that is described as a 3D FE model has to be tracked as a function of time: $V_{c}^{\text{PDE}}(\text{x},t)\;\text{for}\;c\in C$,

While the described approach works for any combination of 3D PDE chambers and 0D ODE chambers, we consider biventricular FE models for our numerical examples in Section 3. This is, the ventricles are modeled as 3D PDEs as in Section 2.2 and the atria are modeled as 0D ODEs described by the *CircAdapt* model as in Appendix A.3. See Fig. 3 for a schematic of this 3D solid–0D fluid coupling.

#### Temporal synchronization of chamber contraction

In the lumped *CircAdapt* model contraction in individual chambers is controlled by prescribed trigger events. Based on the measured heart rate (HR) of 103 beats per minute contraction of the RA was triggered at intervals corresponding to a basic cycle length of 1/HR = 0.585 s. In all other chambers contraction was triggered by prescribed delays relative to the instant of contraction of the RA. In a hybrid coupled model contraction times used in 3D EM (10), (11) and in the lumped *CircAdapt* model (A.8) must be synchronized accordingly. For this sake an interconnected event-driven finite-state machine (FSM) was used to control activation cycles in both 3D and 0D chamber models. Two types of FSMs were used, an autorhythmic FSM to generated triggers at a prescribed cycle length independently of any input, and a reactive excitable FSM of two possible states, excitable or non-excitable. The excitable FSM reacts to external trigger input. If the machine is in excitable state a transition is initiated to the non-excitable state, otherwise, if in non-excitable state, the FSM does not accept the input and remains in a non-excitable state. The FSM returns to its excitable state automatically after a prescribed effective refractory period. A transition from an excitable to a non-excitable state sends out a trigger event to all interconnected FSMs. These interconnections are implemented as delays representing the travel time needed for depolarization wave fronts to propagate to all neighboring interconnected FSMs.

**Figure 4:**
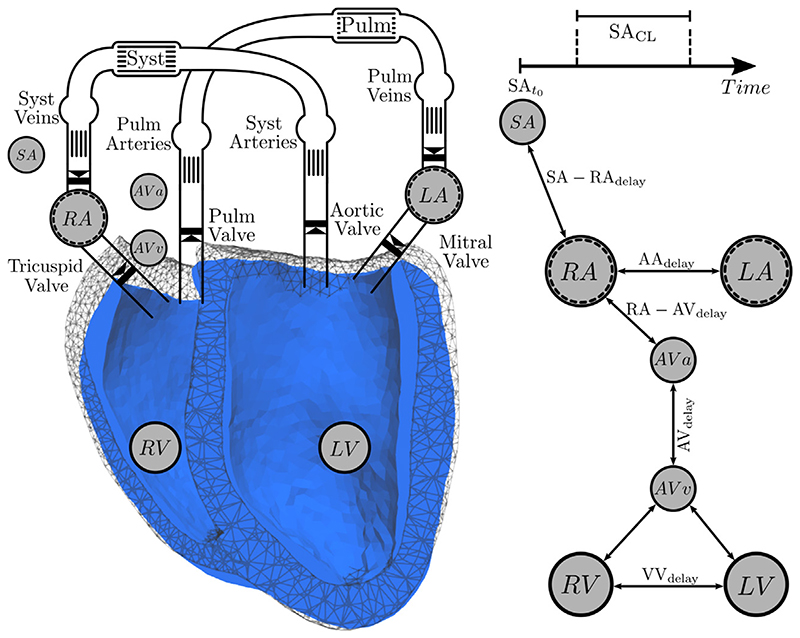
EM activation of the coupled 3D–0D model is steered by an event-driven interconnected FSM that provides triggers for electrical activation of the 3D EM model and for mechanical activation of the 0D lumped atrial cavities. The sino-atrial node clock (SA) activates the RA at a prescribed cycle length, SA_CL_, starting at time SA_t_0__. The LA initiates contraction with a delay of AA_delay_ after the RA. The atrial entrance into the AV node activates at AV_a_ which triggers, after the AV_delay_ elapsed, the ventricular exit of the AV node that is connected to the His bundle at AV_v_. Fascicles in the *LV* are activated then relative to the *LV* trigger to initiate electrical propagation in the EP model. Similarly, the RV is activated with an interventricular delay of VVd before (VV_d_ < 0) or after VV_d_ > 0 the LV.

The triggers provided by the FSM can be flexibly linked to entities within both 3D and 0D model. Specifically, the sino-atrial node is represented by an autorhythmic FSM that is directly interconnected to the RA. Both atrial cavities are implemented as a 0D model that initiate contraction in RA and LA based on FSM trigger events. The RA FSM connects to the atrial entrance to the atrio-ventricular node that transduces excitation through the atrio-ventricular (AV) node with a given delay. The ventricular exit of the AV node is connected to the left and right His bundle that trigger electrical activation of LV and RV. In the LV excitation is initiated by antero-septal, septal and posterior fascicle, and in the RV by a septal and a moderator band fascicle in the RV. As the timing of all fascicles was synchronous between all fascicles of a given chamber, fascicular timings were lumped together under RV and LV (see Fig. 4). RV and LV triggers prescribe fascicular activation times *t*_a_ to the Eikonal equation that

Algorithm 1 **Coupling of the lumped ODE model to the 3D PDE model**

  1: Initialize time *n* = 0

  2: Initialize the set of cavities 𝒞 = {LV, RV, LA, RA}, the set of PDE cavities $C^{\text{PDE}}\subseteq C$, and the set of ODE cavities $C^{\text{ODE}}=C\setminus C^{\text{PDE}}$

  3: Initial displacement ${\underline{u}}_{0}=\underline{0}$

  4: Initial cavity pressures ${\underline{p}}_{C,0}={\left[p_{c}\right]}_{c\in C^{\text{PDE}}}$ at time *n* = 0

  5: Initialize final time point *n*_max_ and maximal number of Newton iterations *k*_max_

  6: Initialize Newton tolerance *ϵ* = 10^−6^

  7: Compute initial cavity volumes ${\underline{V}}^{\text{PDE}}\left({\underline{u}}^{k}\right)={\left[V_{c}^{\text{PDE}}\left({\underline{u}}^{k}\right)\right]}_{c\in C^{\text{PDE}}}$

  8: Run *CircAdapt* ODE system, see Fig. 2, until steady-state is found and get ${\underline{V}}^{\text{ODE}}\left({\underline{p}}_{C,0}\right)={\left[V_{c}^{\text{ODE}}\left(p_{c}\right)\right]}_{c\in C^{\text{PDE}}}$

  9: **while** *n* < *n*_max_ **do**

10:      Initialize Newton iterator: *k* = 0

11:      Initial guesses for Newton: ${\underline{u}}^{0}={\underline{u}}_{n},{\underline{p}}_{C}^{0}={\underline{p}}_{C,n}$

12:      **while** *k* < *k*_max_ **do**

13:          Assemble block matrix                  ▷ Eqs. (B.20)-(B.24) and (C.12)

             and right hand side.               ▷ Eqs. (B.25)-(B.28) and (C.9)-(C.11)

14:          Solve linearized system for $\delta \underline{u}$ and $\delta \underline{p}\underline{c}$                                    ▷ (Eq. B.17)

15:          Update displacement ${\underline{u}}^{k+1}=\underline{u^{k}}+\delta \underline{u}$ and cavity pressures ${\underline{p}}_{C}^{k+1}={\underline{p}}_{C}^{k}+\delta {\underline{p}}_{C}$

16:          Update cavity volumes ${\underline{V}}^{\text{PDE}}\left({\underline{u}}^{k+1}\right)$                                                     ▷ Eq. (14)

17:          Update ODE system and get ${\underline{V}}^{\text{ODE}}\left({\underline{p}}_{C}^{k+1}\right)$                                                ▷ Fig. 2

18:          **Convergence test:**

19:          **if** $\|{\underline{R}}_{\alpha }{\left(\underline{u^{k+1}},{\underline{p}}_{C}^{k+1}\right)}_{{\|}_{L^{2}}}<\epsilon$ **and** $\|{\underline{R}}_{\text{p}}\left({\underline{u}}^{k+1},{\underline{p}}_{C}^{k+1}\right){\|}_{\infty }<\epsilon$ **then**

20:              Solution at time n + 1: ${\underline{u}}_{n+1}=\underline{u^{k+1}},{\underline{p}}_{C,n+1}=\underline{p_{C}^{k+1}}$

21:              **break**                                                             ▷ Newton converged

22:          **else**

23:              *k* = *k* + 1                                                          ▷ Next Newton step

24:          **end if**

25:      **end while**

26:      *n* = *n* + 1                                                                      ▷ Next time step

27: **end while**

governs electrical activation of the ventricular cavities implemented as PDE model. Mechanical contraction of the ventricles was initiated then within a prescribed EM delay, *t*_emd_. The overall concept for synchronizing contraction in the coupled model is illustrated in Fig. 4 and FSM input parameters are given in Table 1.

### 2.6 Parameterization of the baseline model

For the sake of physiological validation the available experimental data were used to calibrate the model in terms of stroke volume (SV) and peak systolic pressure in the LV (${\hat{p}}_{\text{LV}}$). Following [42], initial parameters of passive biomechanics, characterized by the material model given in (8), were taken from [65]; the model was unloaded using a backward displacement algorithm [66] and the material law’s scaling parameter *a* was determined then by fitting the LV model to an empirical Klotz relation [67], using end-diastolic pressure (*p*_ed_) and volume (*V*_ed_) as input. Active stress model parameters τ_c_, *S*_peak_, τ_r_ and duration of the force transient, *t*_dur_ were determined as described previously [25]. Initial values and parameters for the *CircAdapt* model were chosen following [68]. To replicate the observed SV and ${\hat{p}}_{\text{LV}}$ in the left ventricle, input parameters of the active stress model as well as *CircAdapt* model parameters were iteratively adjusted. The final parameterized model beating at 103 bpm produced a cardiac output of ≈2.1 L/min with a SV of 21 mL, in keeping with the experimental data.

### 2.7 Physiological testing

The coupled 3D–0D model was subjected to thorough physiological testing by evaluating its transient response to alterations in loading conditions and contractile state. The model under baseline condition was used as a reference working point relative to which the effect of perturbations in loading and contractility was compared. Standard protocols for assessing of systolic and diastolic properties of the ventricles based on *pV* analysis [69] were implemented to qualitatively gauge the model’s ability to consistently predict known cardiovascular physiology. For all perturbations in preload, afterload, or contractility, two points in time were considered, the immediate acute response after perturbing the system and the new approximate limit cycle reached after 8 beats. For baseline and each limit cycle, the end-systolic pressure volume relation (ESPVR) of the LV was interrogated by imposing additional step changes in afterload. For this sake, end-systolic pressure (*p*_es_) and volume (*V*_es_) were determined in the *pV* loops at the instant of end-systole, as determined by the cessation of flow out of the LV. Linear regression was used then to determine end-systolic elastance, *E*_es_, as the slope of the regression curve, and the volume intercept, *V*_d_, of the ESPVR. Step changes in preload, afterload, and contractility were implemented by varying the cross-sectional area of the pulmonary veins, the systemic vascular resistance, *R*_sys_, and the active peak stress *S*_peak_ generated by the myofilament model, respectively. Overall pump function was also assessed. Following [70], the heart as a pump can be described by the pump function graph (PFG), the relation between mean ventricular pressure, i.e., the ventricular pressure averaged over the entire cardiac cycle, and Cardiac Output. A PFG comprehensively describes cardiac pump function similar to the characterization of industrial pumps and ventricular assist devices. To construct a PFG, data were gathered under all protocols, including additional afterload variations over a wider range, between E_a_ ≈ 0 by setting the system resistance *R*_sys_ ≈ 0 and *E*_a_ ≈ ∞, by closing the aortic valve, to obtain data points under extreme conditions corresponding to the LV beating in absence of external loading and under isovolumetric conditions, respectively.

### 2.8 Numerical framework

After discretization, at each Newton–Raphson step the block system (16) has to be solved. For this sake, we applied a Schur complement approach, see Appendix D, to cast the problem in a pure displacement formulation, to be able to reuse previously established solver methods [41]. In brief, we used the generalized minimal residual method (GMRES) with an relative error reduction of *∈* = 10^−8^. Efficient preconditioning was based on *PETSc* [71] and the incorporated solver suite *hypre*/*BoomerAMG* [72].

The dynamic version of the mechanics equations (3) was also used in other recent studies on cardiac EM [73,74,38] and showed advantages in performance of the linear solver – compared to the more common quasistatic approach – due to a more diagonal-dominant tangent stiffness matrix [75]. For the time integration we used a generalized-*α* scheme, see Appendix C, with spectral radius *𝘱*_∞_ = 0 and damping parameters *β*_mass_ = 0.1 ms^−1^, *β*_stiff_ = 0.1 ms.

We implemented the coupling scheme in the FE framework Cardiac Arrhythmia Research Package (CARPentry) [76,57], built upon extensions of the openCARP EP framework (http://www.opencarp.org). Based on the MATLAB code presented in [44], which is available on the *CircAdapt* website (http://www.circadapt.org), a C++ circulatory system module was implemented into CARPentry to achieve a computationally efficient and strongly scalable numerical scheme that allows fast simulation cycles.

Execution of the 3D–0D model was sped up by limiting the number of Newton steps to *k*_max_ = 1 for the initial series of heart beats that were simulated to stabilize the coupled 3D–0D model to a limit cycle. This corresponds to a semi-implicit (linearly-implicit) discretization method [77] which worked very well in combination with the generalized-*α* scheme. Finally, after arriving at a stable limit cycle two further beats were simulated using a fully converging Newton method with *k*_max_ = 20 and an relative *𝓁*_2_ norm error reduction of the residual of *ϵ* = 10^−6^.

## 3 Results

### 3.1 Parameterization of the baseline model

**Figure 5:**
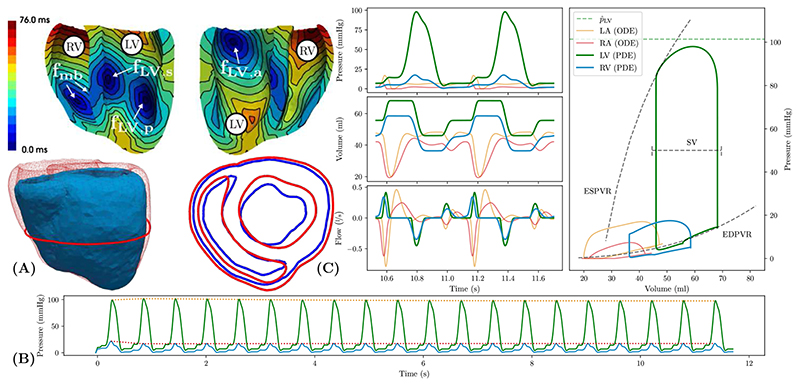
Model parameterization under baseline conditions. (A) The top panels show ventricular sinus activation sequence induced by three LV (f_LV,a_, f_LV,s_, f_LV,p_) and two RV fascicles (f_mb_). The bottom panels show the mechanical end-diastolic (red) and end-systolic (blue) configuration. Note the minor change in epicardial shape due to the pericardial boundary conditions. (B) Simulated pressure traces in LV (green) and RV (blue) are shown for the entire pacing protocol using a train of 20 beats. Envelopes (dotted traces) indicate that an approximate limit cycle was reached after 3 beats. (C) Left panels show time traces of pressure *p*, flow *q* and volume *V* in lumped 0D atrial cavities and PDE-based ventricular cavities for the last two beats of the limit cycle pacing protocol. Variables traverse the state space along limit cycle trajectories. Right panel shows *pV* loops in all four cavities. For PDE-based ventricular cavities EDPVR and ESPVR are indicated. Experimental data on peak LV pressure ${\hat{p}}_{\text{LV}}$ and stroke volume used for fitting are indicated (dashed lines). (For interpretation of the references to color in this figure legend, the reader is referred to the web version of this article.)

The coupled 3D–0D model was fit to approximate the experimental observed data on peak pressure ${\hat{p}}_{\text{LV}}$ and stroke volume in the LV under baseline conditions. Electrical activation was driven by a tri- and bi-fascicular model in LV and RV, respectively, see Fig. 5(A). Conduction velocities were chosen for the given activation pattern to obtain a total ventricular activation time of ~75 ms, compatible with the observed QRS duration of the ECG. Mechanical boundary conditions were set to limit radial contraction of the model, thus leading to a heart beat where ejection was mediated largely by atrio-ventricular plane displacement, i.e. long-axis shortening of the ventricles, and myocardial wall thickening. The resulting end-diastolic and end-systolic configuration of the model is shown in Fig. 5(A). Trains of 20 heart beats were simulated to arrive at an approximate stable limit cycle, as verified by inspecting the slope of the envelope of key hemodynamic state variables, see Fig. 5(C). Corresponding hemodynamic data on pressure, volume, and flows for all four chambers along with the corresponding *pV* loops over the last two beats are shown in Fig. 5(B). Parameter values of the baseline model are given in Tables A.4 and A.5 and Table 2 for the 0D *CircAdapt* and 3D PDE model, respectively.

### 3.2 Effect of spatial resolution

**Figure 6:**
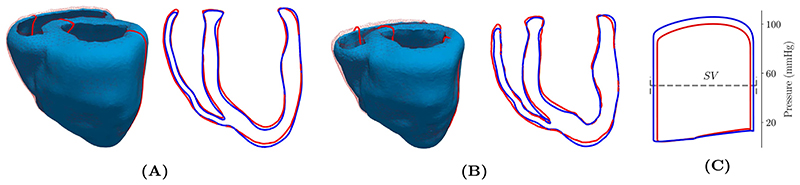
Differences between the coarse (wireframe, red solid line) and higher resolution (solid, blue solid line) model are shown for (A) end-diastolic and (B) end-systolic configuration. (C) Dynamic behavior over the limit cycle protocol was comparable with minor difference in the stroke volume (SV) and peak pressure. (For interpretation of the references to color in this figure legend, the reader is referred to the web version of this article.)

The impact of the relatively coarse spatial resolution (~3.4mm and ~2.4mm for LV and RV, respectively) used was evaluated first by repeating the baseline limit cycle protocol using a higher resolution mesh (~ 1.3mm and ~ 1.2mm for LV and RV, respectively). Both simulations used the exact same set of parameters and initial state vectors. With regard to *pV* behavior that is governed by global deformation of the ventricles, end-diastolic and end-systolic configurations were compared, see Fig. 6. Observed discrepancies between coarse and higher resolution model were marginal and well below the limits of experimental data uncertainty. Differences in ${\hat{p}}_{\text{LV}}$ and SV were less than 5.4% and 6.9%, respectively, suggesting that the computationally efficient coarse model is suitable for performing a physiological validation study.

### 3.3 Physiological testing

**Figure 7:**
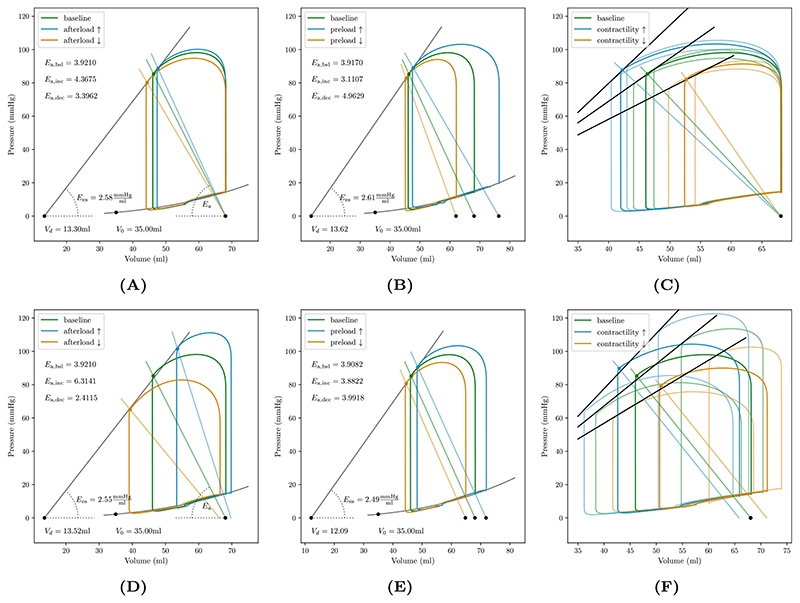
Left ventricular *pV* loops showing the initial response (A–C) and 4 cycles (D–F) after applying a step change in loading conditions and contractility. (A) Altering afterload by increasing/decreasing the systemic vascular resistance, *R*_sys_ pivots arterial elastance *E*_a_ curve. Endsystolic elastance, *E*_es_ and intercept *V*_d_ characterizing the ESPVR was determined by linear regression of end-systolic data points *V*_es_ and *p*_es_, marked by solid circles. (B) Increasing/decreasing preload shifts *E*_a_ curve and increases/decreases stroke volume via the Starling mechanism, mediated by the length-dependence of the active stress model. Determination of ESPVR was consistent with afterload protocol. (C) Increasing/decreasing contractility increases/decreases stroke volume, LV peak pressure and *p*_es_. For each contractile state afterload was also perturbed to determine end-systolic elastance *E*_es_ and *V*_d_.

The response of the coupled 3D–0D system to changes in afterload was probed by altering *R*_sys_ in the range of ±65% around its nominal value of 6350mmHg mL^−1^ ms^−1^ These maneuvers alter the slope of the arterial elastance curve, *E*_a_, pivoting *E*_a_ around the point *V*_ed_ and *p* = 0 in the *pV* diagram. The initial response immediately after step changes in afterload and the new limit cycle are shown in Fig. 7(A) and (D), respectively.

The response to altering LV preload was then probed by stepwise reducing blood flow from the lungs into the LA by varying the cross sectional area of the pulmonary veins. Under such a *walk down* protocol the *E*_a_ curve is shifted to the left towards smaller end-diastolic volumes *V*_ed_, without altering its slope, i.e. *E*_a_ = *p*_es_/SV ≈ const, leading to lower *p*_es_. Stroke volumes under this protocol are assumed to gradually reduce due to the Frank-Starling mechanism, mediated by the length-dependence of active stress generation, *S*_a_(λ). Initial and limit cycle response under this preload perturbation protocol are shown in Fig. 7(B) and (E), respectively. As contractile properties remained unchanged, the same slope *E*_es_ of the ESPVR was obtained as before under step changes in afterload; compare estimated *E*_es_ between Fig. 7(D) and (E).

The effect of step changes in contractility was probed by altering peak active stresses *S*_peak_ in the LV by ±20% around the LV nominal value of 100 kPa This maneuver steepened/flattened the ESPVR, see Fig. 7(C) and (F). In the initial response *V*_es_ and *p*_es_ were affected, with *V*_ed_ remaining constant, this led to a change in stroke volume and an apparent change in arterial elastance estimated by *E*_a_ ≈ *p*_es_/SV, with ≈ −13.09/ + 36.81% relative to baseline. However, in the limit cycle response after readjustment of preload in all chambers, all *E*_a_ curves had the same slope and were only shifted according to the new working *V*_ed_ for the given contractile state. Transient *pV* loops under this protocol are shown in Fig. 7(C) and (F).

**Figure 8:**
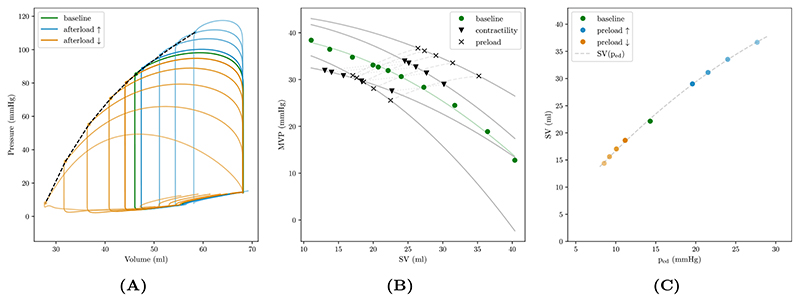
Pump function graph (PFG) and Frank–Starling curve of the LV. (A) *pV* loops in LV under baseline conditions (green) and varying afterload conditions, ranging between unloaded, *E*_a_ ≈ 0, and isometric, *E*_a_ ≈ ∞, conditions. Note that *pV* loops are plotted for the initial beat after altering afterload such that the end-diastolic volume is the same for all conditions. Thus, the system is not in a steady state and *pV* loops are therefore not closed. (B) PFG, plotting mean ventricular pressure (MVP), Eq. (20), against stroke volume (SV), constructed from afterload variations with constant preload and contractility (solid circles), with increased preload (solid squares) shifting the PFG up and left towards higher flow and pressure. For both cases contractility was also perturbed which pivots the PFG (empty circles and squares, respectively), leading to a steeper/flatter slope MVP/ΔSV for increased/decreased contractility. (C) Frank–Starling curve showing the relation between stroke volume and end-diastolic pressure, *SV*(*p*_ed_). (For interpretation of the references to color in this figure legend, the reader is referred to the web version of this article.)

A PFG was constructed by combining data from all tested protocols with additional sampling of data within more extreme ranges of ventriculo-arterial coupling. The models’ PFG is in agreement with known cardiovascular physiology, see Fig. 8. Keeping contractility and preload constant, the PFG with mean ventricular pressure (MVP), as a function of flow or stroke volume can be approximated by a quadratic function, $\text{MVP}(q)={\hat{P}}_{\text{iso}}\left(1-{\left(q/q_{\text{mx}}\right)}^{2}\right)$, with ${\hat{P}}_{\text{iso}}$ and *q*_mx_ being the maximum MVP and maximum flow under isometric and unloaded conditions, respectively. The MVP as a function of time is given by the integral

(20) $$\text{MVP}(t)=\frac{1}{t_{\text{cycle}}}\int_{0}^{t_{\text{cycle}}}p_{\text{LV}}(t+s)\;\text{d}s$$

with a constant cycle length *t*_cycle_. Increasing preload shifts the PFG towards higher flows and pressures. Increasing/decreasing contractility pivots the PFG, leading to a steeper/flatter slope *ΔMVP*/*Δq* of the PFG.

### 3.4 Numerical performance

Computational times for a single heart beat of the lower and higher resolution baseline models are given in Table 3. Simulations were performed on the Vienna Scientific Cluster (VSC4) and we distinguish between solver-time, *t*_s_, which is the accumulated GMRES solver time over all loading/time steps; and assembly-time, *t*_a_, which is the time spent on the setup of boundary conditions and on the assembly of matrices and vectors of the linearized system (16). In total, for a full simulation with loading, 18 initialization beats, and 2 final beats with a fully converging Newton method the computational costs were 4534.38 s for the lower resolution model on 24 cores and 9393.73 s for the higher resolution model on 256 cores. Here, in addition to GMRES solver and assembly times, also the solution of the R-E model governing EP, postprocessing, *CircAdapt* ODE times, and input–output times are taken into account. It is worth noting that the *CircAdapt* ODE solver alone is very efficient: for a simulation of 103 beats (i.e. 1 min with a cycle length of 0.585 s) computational costs were approximately 2 s on one core of a desktop computer. Hence, *CircAdapt* ODE times carry almost no weight in the coupled 3D–0D model.

## 4 Discussion

In this study, we report on the development of a monolithic 3D solid–0D fluid coupling method that allows to flexibly combine 3D PDE and non-linear 0D EM representations of cardiac cavities. The hybrid 3D–0D model of the heart was coupled to a 0D closed-loop model of the cardiovascular system, where all 0D components were based on the *CircAdapt* model [44]. The combined model can be set up to represent one, two, three, or all four cavities as 3D PDE model and all other elements of the cardiovascular system as 0D models based on *CircAdapt*. In this study feasibility of this approach is demonstrated by coupling a 3D PDE model of bi-ventricular EM to 0D model representations of atrial EM function and circulation based on the *CircAdapt* model. The combined model was parameterized under baseline conditions and subjected to comprehensive physiological testing to demonstrate the model’s ability to correctly predict known physiological behaviors. A broad range of experimental protocols for altering loading conditions and contractility were simulated to interrogate the models’ transient responses to these maneuvers [69]. Overall, *pV* analyses of the hemodynamic model output showed close agreement with established knowledge on cardiovascular physiology. The underlying numerical scheme is also represented in detail, including a comprehensive mathematical representation of the *CircAdapt* model. Robustness – in terms of stability and convergence properties – and computational efficiency – in terms of execution times – are demonstrated. These features combined render advanced EM modeling applications feasible. The model facilitates the efficient and robust exploration of parameter spaces over prolonged observation periods which is pivotal for personalizing models to closely match observations. Moreover, the model can be trusted to provide predictions of the acute transient response to interventions or therapies altering loading conditions and contractility that are valid within a commonly accepted physiological reference frame.

## 4.1 Physiological validation

Predictive modeling applications critically rely on the ability of models to encapsulate the most relevant mechanisms governing the cardiovascular response to a given intervention that alters loading conditions or contractility. In a closed-loop cardiovascular system as represented by *CircAdapt*, isolated changes to a single parameter entail transient adaption processes in the system as a whole.

Validation aimed at replicating, overall, known well established behaviors and not at a 1:1 validation against experimental data. For this sake, experimental standard protocols for altering afterload, preload and, contractility were applied to the stabilized baseline model to study its response. Two scenarios were analyzed, the initial acute response to a step change in a single parameter – afterload, preload, or contractility – where effects on other unaltered parameters were minimal, see Fig. 7(A)–(C), and, the limit cycle response observed after a number of beats where transients have largely subsided, but indirect effects led to alteration of other parameters due to the systemic inter-dependencies between these, see Fig. 7(D)–(F).

Afterload was altered by varying the systemic resistance *R*_sys_ and, to mimick more extreme conditions closer to isometric contraction, the resistance of the aortic valve. Increasing/decreasing afterload is reflected in pivoting the arterial elastance curve *E*_a_ around the point of end-diastolic volume and zero pressure. This behavior is illustrated in Fig. 7 and for the more extensive protocols used in constructing the PFG in Fig. 8(A). *E*_a_ was only marginally affected in the initial response, but more significant changes were witnessed after stabilization to a new limit cycle. As expected, changes in slope of *E*_a_ were proportional to changes in *R*_sys_ since, in absence of any regulatory mechanisms, heart rate remained unaltered. Thus, *E*_a_ ≈ MAP/(SV·HR) ≈ *R*_sys_ holds. The phenomenological active stress model as given in Eqs. (10)–(11) accounted for length-dependent tension, but not for velocity dependence. Thus, afterload effects on the velocity of fiber shortening were ignored.

Altering *V*_ed_ by changing the cross section of the pulmonary vein orifices to increase/reduce preload increased/reduced SV. This was due to the Frank–Starling mechanism, as represented by the length–tension relationship of the active stress model in Eq. (11). In the immediate response to a step change in preload the LV emptied to almost the same *V*_es_ with only minor deviations due to changes in arterial pressure induced by the change in SV. After transients subsided, ESPVR and arterial elastance were the same as under baseline conditions, with *E*_a_ being shifted according to the changed *V*_ed_. In the new limit cycle *p*_es_ and MAP were increased, but changes in SV were rather small. This could be attributed to a rather flat slope of the Frank–Starling curve *SV*(*p*_ed_) (not shown) that is related to the significant heterogeneity in fiber stretch in end-diastolic state. Such heterogeneity inevitably arises in biventricular EM models that do not account for residual strains in the unpressurized configuration. As fiber stretch in the reference configuration with *p* = 0 is assumed λ = 1, increasing the filling pressure to *p*_ed_ leads to a significant spread in *λ* around its mean. Thus, while mean λ in our simulations increased with *p*_ed_ the increasing spread of *λ* around its mean led to low fiber stretch λ < λ0 in various regions, particularly in antero-septal and postero-septal segments of the LV. These regions contributed increasingly less to contraction with increasing *p*_ed_ due to the length-dependence of *S*_a_(λ) ≈ 0, thus, leading to an overall attenuation of the Frank–Starling effect.

Altering contractility by increasing/reducing the peak active stress *S*_a_ led to an increase/decrease in SV and systolic pressures in terms of *p* and *p*_es_. Correspondingly, the ESPVR, as sampled by perturbing LV afterload, was steepened/flattened as expected, see Fig. 7(C). In the initial response the slope of *E*_a_ was affected, but after stabilization *E*_a_ was the same for all contractile states. For the given parameterization ventriculo-arterial coupling *E*_es_/*E*_a_ fell outside the optimal range of 1–2, where external work is maximized around *E*_es_/*E*_a_ ≈ 1 and optimal efficiency is achieved at *E*_es_/*E*_a_ ≈ 2. Since $\hat{p}$ and SV and as such *E*_a_ ≈ *p*_es_/SV were used to parameterize the model the resulting slope of the ESPVR was too flat for the LV to operate within this optimal range. Reasons are multifactorial and include the absence of velocity-dependence and, potentially, also the role of mechanical boundary conditions. The main culprit to blame for the limited slope of *E*_es_ is the impairment of the Frank–Starling mechanisms due to fiber stretch heterogeneity. Our model deviates here from the general physiology-based assumption of uniform fiber-stretch to be in place in an end-diastolic state [78–81].

The constructed PFG was qualitatively in keeping with physiological expectations, see Fig. 8. Data points obtained from varying afterload between close to unloaded and isometric conditions agreed well with an assumed quadratic relation between MVP and flow or SV. Increasing preload shifted the PFG up and left towards higher flows and pressures, with the opposite trend being observed for decreasing preload. Altering contractility rotated the PFG.

## 4.2 Numerical aspects

The computational cost imposed by higher resolution EM models requires efficient numerical solvers. Strong scaling characteristics of our numerical framework were reported in detail previously [41,82]. The compute times reported in Section 3.4 indicate that setup and assembly time were the dominating factors during the initial passive inflation (loading) phase while for the subsequent coupled 3D–0D EM simulations of a heart beat solver time was the predominant part of total CPU time. This is due to the Schur complement, see Appendix D, that needs to be solved during the 3D–0D active EM phase. This involved – in our case of a bi-ventricular model comprising two PDE cavities – three applications of the GMRES solver while matrices were only assembled once per Newton step.

Further, significant savings in compute time could be achieved using a semi-implicit approach, see Section 2.6, until a limit cycle was reached. A heartbeat using a fully converging Newton was about five times more expensive compared to a heartbeat in the initialization phase. As the deviations of the semi-implicit method from the implicit scheme were negligible and the final two beats of the limit cycle protocol were then computed using a fully converging Newton method this gain in performance had no quantitative impact on any of the primary simulation outcomes.

Reducing spatial resolution down to 2.4 millimeter and 3.4 millimeter in RV and LV, respectively, allowed for multibeat simulations within tractable time frames using a desktop computer. The impact of this reduction on the hemodynamic output variables was very minor, particularly when viewed in context of the significant observational uncertainties the type of measured data used for model calibration are afflicted with. While probing only two resolutions by far cannot be considered a rigorous convergence study, our results suggest that the use of relatively coarse meshes, with resolutions in the range between 2 to 4mm is adequate. Indeed, this appears to fall within the range of resolutions used in other recent cardiac mechanics simulation studies. For instance, meshes comprising 208 561 [83] and 167 323 [74] tetrahedral elements were used to represent human-sized hearts with all four chambers whereas our coarse model used 45 686 of the same elements only for the ventricles of a much smaller sized canine heart. While our study cannot offer any conclusive recommendations on choosing an appropriate spatial resolution our results suggest that for solving cardiac mechanics problems significantly less spatial resolution is necessary to achieve acceptable accuracy [84] relative to solving EP problems where spatial resolution is critical to resolve the steep propagating depolarization wavefronts [59]. The reaction-Eikonal model we used to represent electrical activation and repolarization is not sensitive to spatial resolution, as shown previously [57], and yields accurate activation patterns on coarse meshes, allowing to use the same grid for both EP and mechanics, without the need for projection of data between the physical grids.

Overall, the total simulation time incurring for 20 heart beats of the coarse model was well below 90min on 24 cores. Similar performance was achieved on a standard desktop computer (AMD Ryzen Threadripper 2990X), demonstrating that realistic multi-beat simulations of the presented 3D–0D cardiac models deliver sufficient performance for advanced physiological simulation scenarios, even on a small number of cores. This is of paramount importance for future parameterization studies where numerous simulations have to be carried out to personalize models to patient data. With around 2 and a half hours on 256 cores for 20 beats also the higher resolution model could be executed within a tractable time frame.

## 4.3 Relation to previous work

The holistic framework described in this study constitutes a major step towards a universal cardiac electromechanics simulation engine that can be applied, in principle and after appropriate parameterization, to a very broad range of applications. Our study builds on and further advances various concepts that have been reported previously in a number of excellent studies [35,33,37,38]. While coupling 3D PDE models to a closed loop circulatory system is important to ensure consistency, by allowing blood to redistribute between compliances in the system, only a few have been reported [37,33,38,40]. However, these were limited in some of the following regards which restricted their universal applicability. For instance, models were discretized with a small number of cubic Hermite elements which led to anatomically stylized representations of the ventricles [85,33,26,86], with an artificially chopped base and a hole or collapsed elements [87] in the apex, owing to their limited ability to accurately accommodate more complex anatomical shapes without greatly increasing computational times [88]. Often, artificial boundary conditions were used that fixed the motion of the base [37,89] and, thus, enforced a zero atrio-ventricular plane displacement. These studies, with only a few exceptions [35,74,90], were unable to replicate a physiological kinematics characterized by the reciprocal filling properties of the heart by maintaining a constant pericardial shape. In other studies EP was not modeled at all, assuming that contraction in the ventricles is initiated simultaneously [38,40,74], or non-physiological activation sequences were used to trigger contraction, both of which impair length-dependent tension mechanisms [37]. Computational cost of numerical methods is not addressed in most previous works; notable exceptions include [37,90,38,74,89] where compute times for one heart beat range between 1.8 and 24 h. This is considerably less efficient compared to compute times presented in this study in Section 3.4. Mostly simplified circuit models were used [37,33,38,40] for simulating a single heart beat or the simulated PV loops featured unphysiological edgy morphologies due to the usage of (i) simple diode valve models not accounting for inertia, Bernoulli effects and flow resistance between compartments and (ii) time-varying elastance models for compliances such as the atria that yield fixed pressure-volume relations and cannot contract in a load-dependent manner as the sarcomere-based contractile 0D chambers used in the *CircAdapt* model.

None of these methodological limitations apply to the modeling framework presented in here.

## 4.4 Limitations

Computational modeling relies on assumptions and approximations, especially multiphysics simulations on organ scale level as presented in this study. While the accuracy of most individual model components was already assessed in previous studies, all these components have limitations and were chosen (i) to achieve great computational performance while preserving almost the full biophysical details of possibly more accurate, computationally costly models; and (ii) to ease parameterization, i.e., to achieve physiological results even with a smaller amount of parameters compared to more accurate but also more complex models. Dependent on the clinical application it might be required to consider other individual components than those chosen in this study.

Efforts to parameterize the combined model were limited, only a small subset of available data were used for model fitting. A generic five-fascicular representation of the cardiac conduction system was used to drive ventricular activation, without attempting to match the recorded ECG [91]. More comprehensive and efficient procedures are required to further enhance compatibility of the high dimensional combined 3D–0D model with all available observations. Building on strategies for fitting the standalone *CircAdapt* model to hemodynamic data [92,93], a hybrid parameterization approach appears a pragmatic solution where the 0D model is fitted first to observed data and in a subsequent parameterization step the 3D cavities are fit to the *pV* characteristics of the corresponding 0D cavities. However, for the sake of demonstrating overall compatibility with known cardiovascular physiology a high fidelity match with experimental data, while desirable, is not crucial. Future more advanced applications that attempt to predict therapeutic responses in a patient-specific manner will critically depend, beyond a comprehensive representation of the most relevant therapy mechanisms, on the fidelity of personalization. Given the large number of model parameters this is not a trivial task that will require the development of dedicated parameter identification strategies. In this regard the outstanding computational performance of the model is essential to facilitate a detailed model personalization which requires a large amount of forward simulations.

While the framework used in this study can be considered universal and feature complete, two important aspects remained unaccounted for. First, active stresses generated by the phenomenological model featured length- but no velocity dependence. However, a velocity-dependent term could be incorporated, as methods that avoid numerical instabilities due to velocity dependence have been reported [73], or a biophysically highly detailed model of excitation–contraction coupling could be used instead as in our previous work [41]. Secondly, residual stresses in the unpressurized ventricles were ignored which impairs, to some extent, the length-dependent Starling effect.

Inflation of the unpressurized configuration to *p*_ed_ without considering residual stresses, inevitably introduces a significant spatial heterogeneity in fiber stretch. This is in contrast to the common assumption of homogeneous fiber stretch in the end-diastolic state [78–81]. This was reflected in a rather flat slope of the Starling curve SV(*p*_ed_). As shown in Fig. 8C, for the given inotropic state and afterload, the Starling curve was close to linear, with a slope of ≈ 1.3mL/mmHg. In humans, slopes in the range between ≈ 2.7–5.5mL/mmHg were measured for non-athletes and athletes, respectively [94].

For the sake of saving computational costs, a coarse spatial resolution was used for discretizing the bi-ventricular model. Thus, models were lightweight enough to carry out the larger number of simulations that were needed for parameterization, the determination of a limit cycle as well as the fine grained testing of physiological maneuvers. The use of such coarse spatial resolutions introduces inaccuracies with regard to fibers and sheet arrangements which are defined on a per element basis. As parts of the model such as the RV wall were composed only of two element layers, transmural fiber rotation was essentially reduced to two fiber families. These were mostly aligned with the endocardial and epicardial fiber orientations as prescribed on a per rule basis, see Fig. 1. Thus, the model’s predictions of motion, strains and stresses may deviate quantitatively from models discretized at higher resolution. However, comparing between two models of different resolution revealed that quantitative differences were minor and, qualitatively, models showed essentially the same behavior. Most differences stemmed from differences in stiffness of the simple P1–P0 element types used which tended to be more compliant for higher resolutions. Nonetheless, errors in simulated hemodynamic outcomes were small enough to be considered negligible when put in context to observational uncertainties clinical or experimental data are afflicted with.

Finally, the presented model is not validated rigorously against experimental data. Typically, an independent validation is performed by the comparison of displacement [95,74,96] and/or strain [55,97,40] to observations from cine MRI or 3D tagged MRI data. However, an accurate cardiac motion and deformation field can be obtained by tuning boundary conditions and *in vivo* MRI strain measurements have major caveats [98,99]. Further, clinical validation can be conducted by comparing simulated ECG, pressure, and volume traces and derived quantities, e.g., SV or ejection fraction, against clinically measured data [25,97,90]. However, commonly, these measurements are all used for model calibration to optimize goodness of fit of simulated outputs to the data and, thus, these cannot be used for the purpose of model validation. Overall, difficulty of proper model validation remains a point of concern and in many cardiac modeling studies validation against actual patient data is limited [100]. In this work, we focused on replicating known, well-established behaviors to show and prove a physiological predictive power under a broad range of experimental protocols. Together with a rigorous, independent validation against image data in future studies, this will set a new standard in cardiac modeling.

## 5 Conclusions

This study reports on a flexible monolithic 3D solid–0D fluid coupling method for integrated models of cardiac EM and cardiovascular hemodynamics. Feasibility of the approach is demonstrated by coupling a 3D PDE model of bi-ventricular EM to 0D model representations of atrial EM and circulation based on the *CircAdapt* model. The coupled 3D–0D model is shown to be robust, computational efficient and able to correctly replicate known physiological behaviors in response to experimental protocols for assessing systolic and diastolic ventricular properties based on *pV* analysis. These features combined render advanced EM modeling applications feasible. The model facilitates the exploration of parameter spaces over prolonged observation periods which is pivotal for personalizing models to closely match observations.

## Supplementary Material

Appendix

## Figures and Tables

**Table 1:** FSM input parameters used for synchronizing electro-mechanical activity comprise heart rate (HR) or cycle length (CL), right atrium (RA), left atrium (LA), intra-atrial (AA), atrio-ventricular (AV) and inter-ventricular (VV) delays and effective refractory period (ERP).

HR	CL	RA	AA delay	AV delay	VV delay	ERP
[beats/min	[s]	[s]	[s]	[s]	[s]	[s]
103	0.585	0.0	0.02	0.1	0.0	0.35

**Table 2:** Input parameters for the 3D PDE model of the left (LV) and right (RV) ventricle. Adjusted to match subject-specific data.

Parameter	Value	Unit	Description
*Passive biomechanics*			
*ρ*_0_	1060.0	kg/m^3^	Tissue density
*κ*	650	kPa	Bulk modulus
*a*	0.7	kPa	Stiffness scaling
*b*_ff_	5.0	[–]	Fiber strain scaling
*b*_ss_	6.0	[–]	Cross-fiber in-plain strain scaling
*b*_nn_	3.0	[–]	Radial strain scaling
*b*_fs_	10.0	[–]	Shear strain in fiber-sheet plane scaling
*b*_fn_	2.0	[–]	Shear strain in fiber-radial plane scaling
*b*_ns_	2.0	[–]	Shear strain in transverse plane scaling
*Active biomechanics*			
*λ*_0_	0.7	ms	Minimum fiber stretch
*V*_m, Thresh_	−60.0	mV	Membrane potential threshold
*t*_emd_	15.0	ms	EM delay
*S*_peak_	100 (LV), 80 (RV)	kPa	Peak isometric tension
*t*_dur_	300.0	ms	Duration of active contraction
*τ* ^c_0_^	100.0	ms	Baseline time constant of contraction
ld	5.0	[–]	Degree of length-dependence
ld_up_	500.0	ms	Length-dependence of upstroke time
*τ*_r_	100.0	ms	Time constant of relaxation
*Electrophysiology*			
*t*_cycle_	0.585	s	Cycle time (= 1/heartrate)
AA delay	20.0	ms	Inter-atrial conduction delay
AV delay	100.0	ms	Atrioventricular conduction delay
VV delay	0.0	ms	Inter-ventricular conduction delay
(*v*_f_, *V*_s_, *V*_n_)	(1.02, 0.68, 0.34)	m/s	Conduction velocities
(*g*_f_, *g*_s_, *g*_n_)	(0.44, 0.54, 0.54)	m/s	Conductivities in LV and RV
*β*	1/1400	cm^-1^	Membrane surface-to-volume ratio
*C*_m_	1	μF/cm^2^	Membrane capacitance

**Table 3:** Summary of numerical metrics for coarse and fine model. Given are the number of compute cores used on VSC4; the average spatial resolution in LV and RV, ${\bar{h}}_{\text{LV}}$ and ${\bar{h}}_{\text{RV}}$; the number of elements and nodes spanning the mesh; as well as solver, assembly, and total times for a single Newton iteration (*t*_s,1_, *t*_a,1_) and a fully converged Newton solution (*t*_s,c_, *t*_a,c_), and the total simulation time per heart beat for single iteration and fully converged Newton scenarios, *T*_b,1_ and *T*_b,c_, respectively. Timings refer to a single heart beat lasting 0.585 s at a time step size of 1 ms. In addition the cumulated solver (*t*_s,ld_) and assembly (*t*_a,ld_) times for the loading phase using 32 load steps are presented.

Model	Cores	${\bar{h}}_{\text{LV}}$/${\bar{h}}_{\text{RV}}$	Elems	Nodes	*t*_s,1_/*t*_a,1_	*t*_s,c_/*t*_a,c_	*T*_b, 1_/*T*_b, c_	*t*_s,ld_/*t*_a,ld_
	[–]	[mm]	[–]	[–]	[s]	[s]	[s]	[s]
Coarse	24	3.4/2.4	45 686	11 850	91.4/42.1	620.2/299.0	135.1/920.8	5.5/14.6
Fine	256	1.3/1.2	557316	111 234	165.6/62.8	1335.8/542.9	231.6/1881.9	12.7/20.6
