# Supplementary material for: A computationally efficient physiologically comprehensive 3D–0D closed-loop model of the heart and circulation
Source: Comput Methods Appl Mech Eng. Author manuscript; Available in PMC 2021 Dec 1. (PMC7611781; doi:10.1016/j.cma.2021.114092)
Supplement: Appendix [file EMS135906-supplement-Appendix.pdf]

Acknowledgments

This project has received funding from the European Union's Horizon 2020 research and innovation programme under the Marie Skłodowska–Curie Action H2020-MSCA-IF-2016 InsiliCardio, GA No.750835 and under the ERA-NET co-fund action No. 680969 (ERA-CVD SICVALVES, JTC2019) funded by the Austrian Science Fund (FWF), Grant I 4652-B to CMA. Additionally, the research was supported by the Grants F3210-N18 and I2760-B30 from the Austrian Science Fund (FWF) and a BioTechMed Graz, Austria flagship award "ILearnHeart" to GP. Further, the project has received funding from the European Union's Horizon 2020 research and innovation programme under the ERA-LEARN co-fund action No. 811171 (PUSHCART, JTC1_27) funded by ERA-NET ERACoSysMed to JL, FWP, EJV, and GP.

Appendix A. *CircAdapt* equations summary

A.1. CircAdapt *tube module*

The tube module represents the entrance of a compliant blood vessel capable of propagating a pressure-flow wave component added to a constant flow, see [27]. Vessels directly attached to the heart, aorta (AO), arteria pulmonalis (AP), venae cavae (VC), and venae pulmonales (VP) are modeled in a similar fashion in *CircAdapt* and for the whole section we define the iterator $t \in \{\text{AO, AP, VC, VP}\}$.

The current lumen cross sectional area is computed by

$$A_t = \frac{V_t}{l_t}, \tag{A.1}$$

with V_t the cavity volume and l_t the length of the vessel segment.

Using a model of a tube with a fibrous wall, see [101], gives the average extension, λ_t, of the fibers in the wall by

$$\lambda_t = \left(1 + 2V_t/V_t^{\text{wall}}\right)^{1/3} = \left(1 + 2A_t/A_t^{\text{wall}}\right)^{1/3},$$

with A_t^{wall} the wall cross-sectional area and $V_t^{\text{wall}} = A_t^{\text{wall}} l_t$ the wall volume. Cavity pressure depends on λ_t

$$p_t = \sigma_t(\lambda_t)\,\lambda_t^{-3},$$

with the mean Cauchy fiber stress σ_t that is modeled by the constitutive equation

$$\sigma_t(\lambda_t) = \sigma_t^{\text{ref}} \cdot \left(\lambda_t/\lambda_t^{\text{ref}}\right)^{k_t},$$

see Arts et al. [101]. Here, k_t a stiffness exponent; $\lambda_t^{\text{ref}} = \left(1 + 2V_t^{\text{ref}}/V_t^{\text{wall}}\right)^{1/3}$ and $\sigma_t^{\text{ref}} = p_t^{\text{ref}}\left(\lambda_t^{\text{ref}}\right)^3$ are the fiber extension and fiber state at normal physiological reference state, respectively; and p_t^{ref} is the reference tube pressure. By combining above results the current tube pressure is computed as

$$\begin{aligned} p_t &= p_t^{\text{ref}}\left(\frac{\lambda}{\lambda_t^{\text{ref}}}\right)^k \lambda_t^{-3}\left(\lambda_t^{\text{ref}}\right)^3 = p_t^{\text{ref}}\left(\frac{\lambda_t}{\lambda_t^{\text{ref}}}\right)^{k-3} \\ &= p_t^{\text{ref}}\left(\frac{V_t^{\text{wall}} + 2V_t}{V_t^{\text{wall}} + 2V_t^{\text{ref}}}\right)^{\frac{k-3}{3}} = p_t^{\text{ref}}\left(\frac{A_t^{\text{wall}} + 2A_t}{A_t^{\text{wall}} + 2A_t^{\text{ref}}}\right)^{\frac{k-3}{3}}, \end{aligned} \tag{A.2}$$

with A_t^{ref} the initial cross sectional area and $V_t^{\text{ref}} = A_t^{\text{ref}} l_t$ the initial vessel volume. The compliance is

$$\begin{aligned} \frac{1}{C_t} = \frac{\mathrm{d}p_t}{\mathrm{d}V_t} &= \frac{\mathrm{d}}{\mathrm{d}V_t}\left[p_t^{\text{ref}}\left(\frac{V_t^{\text{wall}} + 2V_t}{V_t^{\text{wall}} + 2V_t^{\text{ref}}}\right)^{\frac{k-3}{3}}\right] \\ &= p_t^{\text{ref}}\,\frac{k-3}{3}\left(\frac{V_t^{\text{wall}} + 2V_t}{V_t^{\text{wall}} + 2V_t^{\text{ref}}}\right)^{\left(\frac{k-3}{3}-1\right)} \frac{2}{V_t^{\text{wall}} + 2V_t^{\text{ref}}} \\ &= \frac{2p_t\,(k-3)}{3\left(V_t^{\text{wall}} + 2V_t\right)} = \frac{2p_t\,(k-3)}{3l_t\left(A_t^{\text{wall}} + 2A_t\right)}. \end{aligned}$$

Finally, the basic relation between characteristic wave impedance Z_t compliance C_t, and inertance I_t

$$Z_t^2 = \frac{I_t}{C_t} = \frac{\rho_\mathrm{b} l_t}{A_t C_t} \tag{A.3}$$

yields

$$Z_t = \sqrt{\frac{2\rho_\mathrm{b}\, p_t\, l_t^2(k-3)}{3V_t\left(V_t^\mathrm{wall}+2V_t\right)}} = \sqrt{\frac{2\rho_\mathrm{b}\, p_t\,(k-3)}{3A_t\left(A_t^\mathrm{wall}+2A_t\right)}} \tag{A.4}$$

with ρ_b the blood density.

A.2. Sarcomere mechanics

In the following a sarcomere contraction model is described that is based on a modified Hill model, see [61,44], for all tissue patches in the wall of the cavity: $c \in \{\mathrm{LV}, \mathrm{RV}, \mathrm{Sep}, \mathrm{LA}, \mathrm{RA}\}$, with the left (LV) and right (RV) ventricle, the septum (Sep), and the left (LA) and right (RA) atrium. Natural strain E_c^fib of the myofiber is estimated as

$$E_c^\mathrm{fib} = \ln\left(\frac{L_c^\mathrm{s}}{L^\mathrm{s,ref}}\right) \tag{A.5}$$

and from this the total sarcomere length L_c^s can be computed as

$$L_c^\mathrm{s} = L^\mathrm{s,ref}\exp\left(E_c^\mathrm{fib}\right), \tag{A.6}$$

with $L_c^\mathrm{s,ref}$ a constant describing the reference sarcomere length. The sarcomere is supposed to be made up of a contractile element of length L_c^cont in series with an elastic element of length $L_c^\mathrm{elast} = L_c^\mathrm{s} - L_c^\mathrm{cont}$.

Sarcomere active stress

Sarcomere contracting length L_c^cont varies over time according to

$$\dot{L}_c^\mathrm{cont} := \frac{\mathrm{d}L_c^\mathrm{cont}}{\mathrm{d}t} = v^\mathrm{max}\left[\frac{L_c^\mathrm{s} - L_c^\mathrm{cont}}{L^\mathrm{elast,iso}} - 1\right], \tag{A.7}$$

where the constant $L^\mathrm{elast,iso}$ is the length of the series elastic element during isovolumetric contraction and the constant v^max is the maximum velocity of contraction.

The governing equation for the contractility C_c, describing the density of cross bridge formation within the fibers of the patch, is

$$\dot{C}_c := \frac{\mathrm{d}C_c}{\mathrm{d}t} = f_c^\mathrm{rise}(t) C_c^\mathrm{s}\left(L_c^\mathrm{cont}\right) - f_c^\mathrm{decay}(t) C_c. \tag{A.8}$$

In Eq. (A.8) sarcomere contractility rises according to the function f_c^rise, a phenomenological representation of the rate of cross bridge formation within the patch,

$$f_c^\mathrm{rise}(t) = \frac{1}{t_c^\mathrm{rise}} 0.02 x_c^3 (8 - x_c)^2 \exp(-x_c),$$

$$x_c(t) = \min\left(8, \max\left(0, \frac{t - t_c^\mathrm{act}}{t_c^\mathrm{rise}}\right)\right),$$

depending on the time of onset of activation of the patch, t_c^act, and the rising time

$$t_c^\mathrm{rise} = 0.55\tau^\mathrm{R} t_c^\mathrm{act,ref}.$$

Here, τ^R a constant and $t_c^\mathrm{act,ref}$ is the reference duration of contraction for initial fiber length.

Sarcomere contractility in (A.8) decays according to the function f_c^decay

$$f_c^\mathrm{decay}(t) = \frac{1}{2t_c^\mathrm{decay}}\left[1 + \sin\left(\mathrm{sign}(y_c)\min\left(\frac{\pi}{2}, |y_c|\right)\right)\right],$$

$$y_c(t) = \frac{t - t_c^{\text{act}} - t_c^{\text{act,dur}}}{t_c^{\text{decay}}},$$

depending on the decay time

$$t_c^{\text{decay}} = 0.33\tau^{\text{D}} t_c^{\text{act,ref}},$$

with τ^{D} a constant and $t_c^{\text{act,dur}}$ is the duration of contraction of the fiber that lengthens with sarcomere length

$$t_c^{\text{act,dur}} = \left(0.65 + 1.0570 L_c^{\text{norm}}\right) t_c^{\text{act,ref}}.$$

Here, L_c^{norm} is the normalized sarcomere length for active contraction

$$L_c^{\text{norm}} = \max\left(0.0001, L_c^{\text{cont}}/L^{\text{act0,ref}} - 1\right),$$

where $L^{\text{act0,ref}}$ is the zero active stress sarcomere length.

C_c^{s} in (A.8) describes the increase in cross bridge formation with intrinsic sarcomere length due to an increase in available binding sites,

$$C_c^{\text{s}}\left(L_c^{\text{cont}}\right) = \tanh\left(0.75 * 9.1204\left(L_c^{\text{norm}}\right)^2\right).$$

Contractility C_c (A.8) and sarcomere contracting length L_c^{cont} (A.7) are used to compute the actively generated fiber stress

$$\sigma_c^{\text{fib,act}} = L^{\text{act0,ref}} \sigma^{\text{act,max}} C_c L_c^{\text{norm}} \frac{L_c^{\text{s}} - L_c^{\text{cont}}}{L^{\text{elast,iso}}}, \tag{A.9}$$

with constants $L^{\text{act0,ref}}$, $\sigma^{\text{act,max}}$, $L^{\text{elast,iso}}$, see Table A.5.

Sarcomere passive stress

Passive stress $\sigma_c^{\text{fib,pas}}$ is considered to contain two components,

$$\sigma_c^{\text{fib,pas}} = \sigma_c^{\text{fib,tit}} + \sigma_c^{\text{fib,ecm}}, \tag{A.10}$$

first the stress arising from cellular structures such as titin, a highly abundant structural protein of the sarcomere, anchoring to the Z-disc, $\sigma_c^{\text{fib,tit}}$, and second the stress arising from the extracellular matrix (ECM), $\sigma_c^{\text{fib,ecm}}$. Both depend on the passive fiber stretch which is computed as

$$\lambda_c^{\text{pas}} = \frac{L_c^{\text{s}}}{L^{\text{pas0,ref}}},$$

where $L^{\text{pas0,ref}}$ is sarcomere length with zero passive stress and L_c^{s} the total sarcomere length, see above. Using that we compute

$$\sigma_c^{\text{fib,tit}} = 0.01\sigma^{\text{act,max}}\left(\left[\lambda_c^{\text{pas}}\right]^{k^{\text{tit}}} - 1\right),$$

with $\sigma^{\text{act,max}}$ the maximal isometric stress and the constant exponent

$$k^{\text{tit}} = 2\frac{L^{\text{s,ref}}}{\text{d}L^{\text{s,pas}}}.$$

The ECM is modeled as being stiffer than the myocyte contribution using

$$\sigma_c^{\text{fib,ecm}} = 0.0349\sigma^{\text{pas,max}}\left(\left(\lambda_c^{\text{pas}}\right)^{10} - 1\right),$$

where $\sigma^{\text{pas,max}}$ is an empirical parameter.

Sarcomere total stress

Total myofiber stress σ_c^{fib} is the sum of an active (A.9) and a passive (A.10) stress component

$$\sigma_c^{\text{fib}} = \sigma_c^{\text{fib,act}} + \sigma_c^{\text{fib,pas}}. \tag{A.11}$$

Sarcomere stiffness κ_c^{fib} is now computed as the derivative of total fiber stress (A.11) with respect to fiber strain (A.5)

$$\kappa_c^{\text{fib}} = \frac{\partial \sigma_c^{\text{fib}}}{\partial E_c^{\text{fib}}} = \frac{\partial \sigma_c^{\text{fib,act}}}{\partial E_c^{\text{fib}}} + \frac{\partial \sigma_c^{\text{fib,pas}}}{\partial E_c^{\text{fib}}}, \tag{A.12}$$

with

$$\frac{\partial \sigma_c^{\text{fib,act}}}{\partial E_c^{\text{fib}}} = L^{\text{act0,ref}} \sigma^{\text{act,max}} C_c L_c^{\text{norm}} \frac{L_c^{\text{s}}}{L^{\text{elast,iso}}},$$

$$\frac{\partial \sigma_c^{\text{fib,pas}}}{\partial E_c^{\text{fib}}} = 0.01 k^{\text{tit}} \sigma_c^{\text{act,max}} \left(\lambda_c^{\text{pas}}\right)^{k^{\text{tit}}} + 0.0349 * 10 \sigma^{\text{pas,max}} \left(\lambda_c^{\text{pas}}\right)^{10}.$$

A.3. CircAdapt *chamber module*

An actively contracting chamber $c \in \{\text{LV}, \text{RV}, \text{LA}, \text{RA}\}$ is modeled using the state variables volume V_c, length of the contractile element of the sarcomere L_c^{cont} (A.7), and contractility C_c (A.8). Volume changes driven by inflow and outflow of blood induce changes in midwall volume V_c^{mid} and area A_c^{mid}.

Sphere mechanics

Note that in *CircAdapt* ventricles are usually modeled using the TriSeg formulation, see Appendix A.4. If TriSeg is turned on, the calculations in this chapter are only used for the atria while ventricular values are computed as in Appendix A.4.

Midwall volume V_c^{mid} is estimated as

$$V_c^{\text{mid}} = V_c + \frac{1}{2} V_c^{\text{wall}}, \tag{A.13}$$

where V_c^{wall} is constant wall volume. If not set to a specific value the wall volume is estimated by extruding the sphere enclosing the cavity volume V_c by a constant wall thickness h_c^{wall}, see Table A.4. Chambers are modeled as closed spheres, thus, the following equations result from volume and surface formulas for spheres

$$C_c^{\text{mid}} = \left(\frac{4\pi}{3 V_c^{\text{mid}}}\right)^{1/3}, \tag{A.14}$$

$$A_c^{\text{mid,tot}} = \frac{4\pi}{\left(C_c^{\text{mid}}\right)^2}, \tag{A.15}$$

$$A_c^{\text{mid}} = A_c^{\text{mid,tot}} - A_c^{\text{mid,dead}}, \tag{A.16}$$

where C_c^{mid} is midwall curvature, i.e., the inverse of radius; and $A_c^{\text{mid,dead}}$ is non-contractile area, i.e., valve openings and orifices.

Update fiber strain

Natural fiber strain E_c^{fib} is calculated by

$$E_c^{\text{fib}} = \frac{1}{2} \ln \left(\frac{A_c^{\text{mid}}}{A_c^{\text{mid,ref}}}\right) \tag{A.17}$$

with $A_c^{\text{mid,ref}}$ the surface area in the reference state, see [44]. Note that this updated fiber strain is used in the place of (A.5) to update values in the sarcomere module Appendix A.2.

Cross-sectional area A_c of chambers is estimated as

$$A_c = \frac{V_c + 0.1 V_c^{\text{wall}}}{l_c}, \tag{A.18}$$

$$l_c = 2\left(V_c^{\text{mid}}\right)^{1/3},$$

with l_c the long-axis length of the cavity.

The characteristic wave impedance Z_c is approximated according to (A.3), see also [27], and by applying the chain rule

$$Z_c = \frac{1}{5A_c}\sqrt{\rho_b l_c \left|\kappa_c^{\mathrm{mid}}\right|}, \tag{A.19}$$

with the sheet stiffness

$$\kappa_c^{\mathrm{mid}} = \frac{\partial T_c^{\mathrm{mid}}}{\partial A_c^{\mathrm{mid}}} = \frac{V_c^{\mathrm{wall}}}{4\left(A_c^{\mathrm{mid}}\right)^2}\left(\frac{\partial \sigma_c^{\mathrm{fib}}}{\partial E_c^{\mathrm{fib}}} - 2\sigma_c^{\mathrm{fib}}\right) = \frac{V_c^{\mathrm{wall}}}{4\left(A_c^{\mathrm{mid}}\right)^2}\left(\kappa_c^{\mathrm{fib}} - 2\sigma_c^{\mathrm{fib}}\right) \tag{A.20}$$

and the updated fiber stiffness κ_c^{fib}, see Eq. (A.12).

Conservation of energy

CircAdapt connects midwall tension T_c^{mid} and midwall area A_c^{mid} to fiber stress σ_c^{fib} and strain E_c^{fib} through the law of conservation of energy. With the law of Laplace we get

$$T_c^{\mathrm{mid}}\,\mathrm{d}A_c^{\mathrm{mid}} = \sigma_c^{\mathrm{fib}} V_c^{\mathrm{wall}}\,\mathrm{d}E_c^{\mathrm{fib}} \tag{A.21}$$

and with (A.17) we get for the midwall tension

$$T_c^{\mathrm{mid}} = \frac{\sigma_c^{\mathrm{fib}} V_c^{\mathrm{wall}}}{2A_c^{\mathrm{mid}}}. \tag{A.22}$$

Transmural pressure p_c^{trans} is finally computed as follows

$$p_c^{\mathrm{trans}} = 2T_c^{\mathrm{mid}} C_c^{\mathrm{mid}}. \tag{A.23}$$

Since at the moment external pressures are assumed to be zero, the transmural pressure coincides with the internal pressure of the contracting chamber

$$p_c = p_c^{\mathrm{trans}}.$$

A.4. TriSeg model of ventricular interaction

In case that one ODE and one PDE cavity is included in the model, ventricles are modeled as atria above. Otherwise, ventricular and septal midwall volumes are modeled as a ventricular composite [61] which is defined by the common radius y^{mid} of the wall junction and the enclosed midwall cap volumes, see Fig. A.9c. Midwall cap volumes of the right and the left ventricle are computed as

$$V_{\mathrm{LV}}^{\mathrm{mid}} = -V_{\mathrm{LV}} + V_{\mathrm{Sep}}^{\mathrm{mid}} - \frac{1}{2}\left(V_{\mathrm{LV}}^{\mathrm{wall}} + V_{\mathrm{Sep}}^{\mathrm{wall}}\right),$$

$$V_{\mathrm{RV}}^{\mathrm{mid}} = V_{\mathrm{RV}} + V_{\mathrm{Sep}}^{\mathrm{mid}} + \frac{1}{2}\left(V_{\mathrm{RV}}^{\mathrm{wall}} + V_{\mathrm{Sep}}^{\mathrm{wall}}\right).$$

Here, the wall volumes of the left, $V_{\mathrm{LV}}^{\mathrm{wall}}$, and right, $V_{\mathrm{RV}}^{\mathrm{wall}}$, ventricle are constants. The blood pool volumes of the left, V_{LV}, and right, V_{RV}, ventricle are ODE variables as well as the radius y^{mid} and the septal midwall volume $V_{\mathrm{Sep}}^{\mathrm{mid}}$. Note that the sign of midwall volume V_c^{mid} is positive if wall curvature is convex to the positive x-direction and negative otherwise.

Distance x_c^{mid}, see Fig. A.9c, is then computed by the relation

$$V_c^{\mathrm{mid}} = \frac{\pi}{6} x_c^{\mathrm{mid}}\left((x_c^{\mathrm{mid}})^2 + 3(y^{\mathrm{mid}})^2\right), \quad \text{for } c \in \{\mathrm{LV}, \mathrm{RV}, \mathrm{Sep}\},$$

hence

$$x_c^{\mathrm{mid}} = q_c - \frac{(y^{\mathrm{mid}})^2}{q_c}, \text{ with } q_c = \sqrt[3]{\sqrt{\left(\frac{3}{\pi}V_c^{\mathrm{mid}}\right)^2 + \left(y^{\mathrm{mid}}\right)^6} + \frac{3}{\pi}V_c^{\mathrm{mid}}}.$$

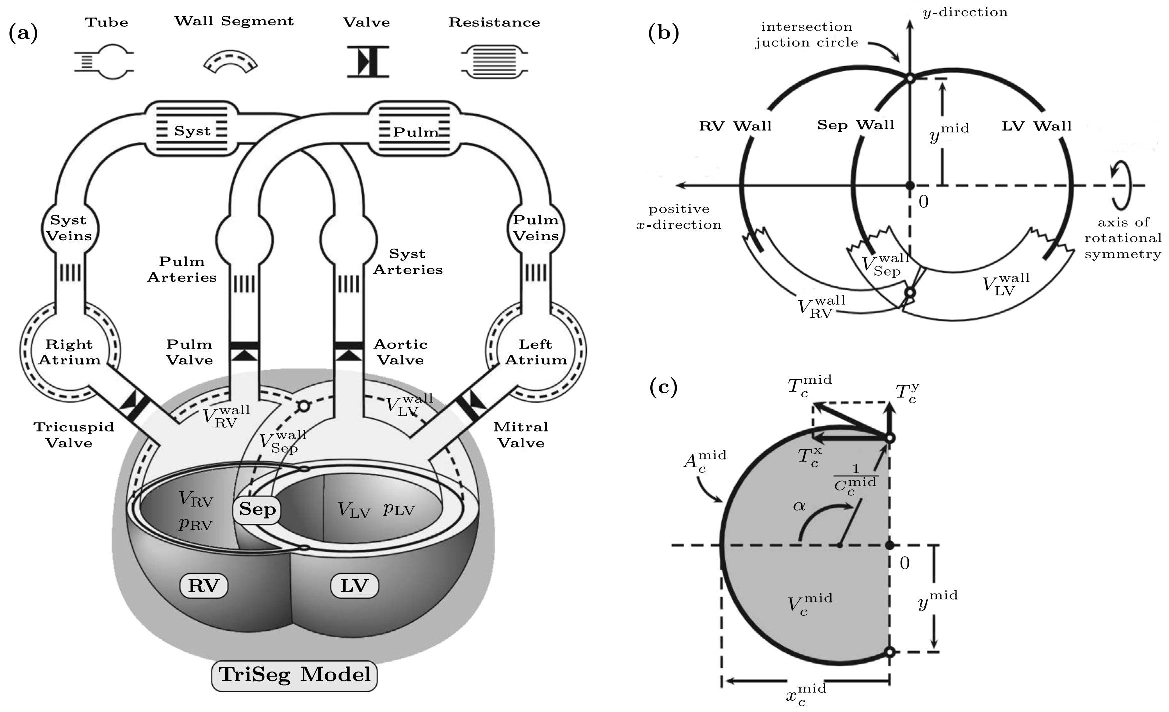

Fig. A.9. TriSeg model of septal (Sep) and left (LV) and right ventricular (RV) mechanics. (a) The TriSeg model (gray shading) incorporated in the modular *CircAdapt* model of the systemic (Syst) and pulmonary (Pulm) circulations. (b) Cross-section of the ventricular composite. (c) Cross-section of a single wall segment ($c \in \{\mathrm{LV}, \mathrm{RV}, \mathrm{Sep}\}$) through the axis of rotational symmetry.
Source: Adapted with permission from [61].

Midwall area and curvature are consequently computed

$$A_c^{\mathrm{mid}} = \pi \left((x_c^{\mathrm{mid}})^2 + (y^{\mathrm{mid}})^2 \right), \qquad \text{for } c \in \{\mathrm{LV}, \mathrm{RV}, \mathrm{Sep}\},$$

$$C_c^{\mathrm{mid}} = \frac{2x_c^{\mathrm{mid}}}{(x_c^{\mathrm{mid}})^2 + (y^{\mathrm{mid}})^2}, \qquad \text{for } c \in \{\mathrm{LV}, \mathrm{RV}, \mathrm{Sep}\},$$

and used to calculate midwall tension T_c^{mid} (A.22).

Axial T_c^{x} and radial T_c^{y} tension components are computed using laws of trigonometry

$$T_c^{\mathrm{x}} = T_c^{\mathrm{mid}} \sin\alpha, \quad \text{with } \sin\alpha = \frac{2x_c^{\mathrm{mid}}\, y^{\mathrm{mid}}}{(x_c^{\mathrm{mid}})^2 + (y^{\mathrm{mid}})^2}, \qquad \text{for } c \in \{\mathrm{LV}, \mathrm{RV}, \mathrm{Sep}\},$$

$$T_c^{\mathrm{y}} = T_c^{\mathrm{mid}} \cos\alpha, \quad \text{with } \cos\alpha = \frac{-(x_c^{\mathrm{mid}})^2 + (y^{\mathrm{mid}})^2}{(x_c^{\mathrm{mid}})^2 + (y^{\mathrm{mid}})^2}, \qquad \text{for } c \in \{\mathrm{LV}, \mathrm{RV}, \mathrm{Sep}\}.$$

It is required that the total midwall tension at junctions is zero, i.e.,

$$f(y^{\mathrm{mid}}, V_{\mathrm{Sep}}^{\mathrm{mid}}) := \begin{pmatrix} T_{\mathrm{LV}}^{\mathrm{x}} + T_{\mathrm{RV}}^{\mathrm{x}} + T_{\mathrm{Sep}}^{\mathrm{x}} \\ T_{\mathrm{LV}}^{\mathrm{y}} + T_{\mathrm{RV}}^{\mathrm{y}} + T_{\mathrm{Sep}}^{\mathrm{y}} \end{pmatrix} \stackrel{!}{=} 0. \tag{A.24}$$

Eq. (A.24) is solved by an iterative Newton scheme

$$f'(y_k^{\mathrm{mid}}, V_{k,\mathrm{Sep}}^{\mathrm{mid}}) \left(\Delta y_k^{\mathrm{mid}}, \Delta V_{k,\mathrm{Sep}}^{\mathrm{mid}} \right)^\top = -f(y_k^{\mathrm{mid}}, V_{k,\mathrm{Sep}}^{\mathrm{mid}}), \quad k = 1, 2, \ldots \tag{A.25}$$

and increments Δy_k^{mid} and $\Delta V_{k,\text{Sep}}^{\text{mid}}$ are added to y_k^{mid} and $V_{k,\text{Sep}}^{\text{mid}}$. The solution of (A.25) in the first step, i.e., for $k = 0$ is used to define the ODE updates for the septum

$$\dot{V}_{\text{Sep}}^{\text{mid}} = \frac{1}{\tau_{\text{Sep}}} \Delta V_{0,\text{Sep}}^{\text{mid}}, \quad \dot{y}^{\text{mid}} = \frac{1}{\tau_{\text{Sep}}} \Delta y_0^{\text{mid}}, \tag{A.26}$$

where τ_{Sep} is a time constant.

Consequently, the values for the tensions discussed above are updated and the scheme is iterated until convergence. Midwall volumes are updated by

$$V_c^{\text{mid}} = V_c + \frac{1}{2}\left(V_c^{\text{wall}} + V_{\text{Sep}}^{\text{wall}}\right),$$

long-axis length l_c and cross-sectional area A_c and of the cavity are computed by

$$l_c = 2\left(V_c^{\text{mid}} + \frac{1}{2}\left(V_c^{\text{wall}} + V_{\text{Sep}}^{\text{wall}}\right)\right)^{1/3}, \tag{A.27}$$

$$A_c = \frac{V_c^{\text{mid}} + \frac{1}{20}\left(V_c^{\text{wall}} + V_{\text{Sep}}^{\text{wall}}\right)}{l_c}. \tag{A.28}$$

Finally, wave impedance Z_c is computed according to Eq. (A.19) and transmural pressure p_c^{trans} is computed as total axial force

$$p_c^{\text{trans}} = 2\,\frac{T_c^{\text{x}}}{y^{\text{mid}}}, \quad \text{for } c \in \{\text{LV}, \text{RV}, \text{Sep}\}.$$

Assuming the pressure surrounding the ventricular composite to be zero, internal chamber pressure for the ventricles is now found as

$$p_{\text{LV}} = -p_{\text{LV}}^{\text{trans}},$$
$$p_{\text{RV}} = p_{\text{RV}}^{\text{trans}}.$$

A.5. Pericardial mechanics

The four cardiac chambers are supposed to have an additional pressure component due to the pericardium. Pressure p_{peri} exerted by the pericardial sack on atria and ventricles was computed as a non-linear function of pericardial volume V_{peri}, computed as the sum of blood pool and wall volumes of the four cardiac chambers:

$$V_{\text{peri}} = V_{\text{LV}} + V_{\text{RV}} + V_{\text{LA}} + V_{\text{RA}} + V_{\text{LV}}^{\text{wall}} + V_{\text{RV}}^{\text{wall}} + V_{\text{LA}}^{\text{wall}} + V_{\text{RA}}^{\text{wall}} \tag{A.29}$$

$$p_{\text{peri}} = p_{\text{peri}}^{\text{ref}}\left(\frac{V_{\text{peri}}}{V_{\text{peri}}^{\text{ref}}}\right)^{k_{\text{peri}}}, \tag{A.30}$$

where $p_{\text{peri}}^{\text{ref}}$ and $V_{\text{peri}}^{\text{ref}}$ are constant reference pressure and volume, respectively, and k_{peri} defines the degree of non-linearity of the pressure–volume relation.

Cavity pressures are updated according to

$$p_c = p_c + p_{\text{peri}}, \quad \text{for } c \in \{\text{LV}, \text{RV}, \text{LA}, \text{RA}\}. \tag{A.31}$$

A.6. Periphery

Pulmonary (pulm) and systemic (sys) periphery are modeled as resistances. The current pressure drop Δp_{py}, for $py \in \{\text{pulm}, \text{sys}\}$, is computed as the difference of the pressures in the inflow artery p_t^{prox} and the outflow vein p_t^{dist}:

$$\Delta p_{py} = p_t^{\text{prox}} - p_t^{\text{dist}}.$$

Using this, the current flow over the periphery is

$$q_{py} = q_{py}^{\text{ref}} \left(r_{py} \frac{\Delta p_{py}}{\Delta p_{py}^{\text{ref}}} \right)^{k_{py}}, \tag{A.32}$$

where $\Delta p_{py}^{\text{ref}}$ is the reference arteriovenous pressure drop; q_{py}^{ref} is the reference flow over the periphery; r_{py} is a scaling factor of the arteriovenous resistances; and k_{py} is a factor that accounts for the nonlinearity of the arteriovenous resistances, see Tables A.4 and A.5.

A.7. Connect modules

Volume change of inflow arteries $\dot{V}_t^{\text{prox}}$ and outflow veins $\dot{V}_t^{\text{dist}}$ is now updated by

$$\begin{aligned} \dot{V}_t^{\text{dist}} &\mathrel{+}= q_{py} \\ \dot{V}_t^{\text{prox}} &\mathrel{+}= q_{py} \end{aligned} \tag{A.33}$$

Computation of time derivative of flow across valves and venous-atrial inlet requires as input the cross-sectional area of proximal and distal elements to the channel.

$$\begin{aligned} \dot{V}_{c,t}^{\text{dist}} &\mathrel{+}= q_v \\ \dot{V}_{c,t}^{\text{prox}} &\mathrel{+}= q_v \end{aligned} \tag{A.34}$$

$$\begin{aligned} p_{c,t}^{\text{prox}} &\mathrel{+}= \dot{V}_{c,t}^{\text{prox}} Z_{c,t}^{\text{prox}} \\ p_{c,t}^{\text{dist}} &\mathrel{+}= \dot{V}_{c,t}^{\text{dist}} Z_{c,t}^{\text{dist}} \end{aligned} \tag{A.35}$$

A.8. Valve dynamics

The pressure drop (Δp_v) across a valve is the sum of the effects of inertia due to acceleration in time and the Bernoulli effect, see [102]

$$\Delta p_v = \rho_{\text{b}} \frac{l_v}{A_v} \dot{q}_v + \frac{\rho_{\text{b}}}{2} \left((v_v^{\text{out}})^2 - (v_v^{\text{in}})^2 \right), \tag{A.36}$$

where ρ_{b} is the density of blood, A_v is the current cross-sectional area of the valve, and l_v is the length of the channel with inertia. If not mentioned otherwise this value is estimated as

$$l_v = \sqrt{A_v^{\text{open}}},$$

with A_v^{open} the given cross-sectional area of the open valve, see Table A.4. For $q_v \geq 0$, v_v^{in} is the velocity proximal to the valve v_v^{prox}. v_v^{out} is the maximum of the blood velocities in the valve region $v_v^{\max} = \max(v_v^{\text{dist}}, v_v, v_v^{\text{prox}})$. For $q_v < 0$ which indicates that the valve is leaking v_v^{in} is the velocity distal to the valve v_v^{dist} and the outflow velocity is the maximum of the blood velocities in the valve region $v_v^{\text{out}} = v_v^{\max}$. Using $v_v = q_v/A_v$ we can write

$$\Delta p_v = p_v^{\text{prox}} - p_v^{\text{dist}} = \alpha_v \dot{q}_v + \beta_v q_v^2 \tag{A.37}$$

with

$$\alpha_v = \rho_{\text{b}} \frac{l_v}{A_v} \tag{A.38}$$

the inertia of the channel. The open/closed status of the valve is a function of pressure drop and flow. Valves are clearly open/closed if both pressure drop and flow point in the same direction. With forward pressure drop, the valve opens immediately. With backward pressure and forward flow, the valve is closing softly by a continuous function

$$\begin{aligned} A_v^{\text{closing}} &= \sqrt{\frac{x_v}{x_v^2 + \Delta p_v^2}} \left(A_v^{\text{open}} - A_v^{\text{leak}} \right) + A_v^{\text{leak}} \\ x_v &= \frac{40 \rho_{\text{b}} \, q_v \, |q_v|}{\left(A_v^{\text{open}} \right)^2}, \end{aligned} \tag{A.39}$$

Table A.4
Input parameters for the *CircAdapt* model. Adjusted to match patient-specific data.

Parameter	Value	Unit	Description
General			
ρ_b	1050.0	kg/m^3	Blood density
t_cycle	0.585	s	Cycle time (= 1/heartrate)
Tubes: aorta (AO), arteria pulmonalis (AP), venae cavae (VC), and venae pulmonales (VP)			
$A_\mathrm{t}^\mathrm{wall}$	274 (AO), 141 (AP), 58 (VC), 85 (VP)	mm^2	Cross-sectional wall area
l_t	500 (AO), 400 (VC), 200 (AP, VP)	mm	Length of vessel
$A_\mathrm{t}^\mathrm{ref}$	Adjacent valve area	mm^2	Initial cross sectional area
k_t	5 (AO), 8 (AP), 10 (VC, VP)	[–]	Stiffness exponent
Chambers: left (LV) and right (RV) ventricle; left (LA) and right atrium (RA)			
V_c	57.0 (LV), 75.3 (RV), 44.2 (LA), 54.4 (RA)	mL	Cavity volume
$h_\mathrm{c}^\mathrm{wall}$	15.0 (LV), 4.0 (RV), 2.0 (LA), 2.0 (RA)	mm	Constant wall thickness
$\Delta t_\mathrm{c}^\mathrm{act}$	0.1 (LV), 0.1 (RV), 0.02 (LA), 0.0 (RA)	s	Delays of onset of activation in each beat starting at t^bt; $t_c^\mathrm{act} = t^\mathrm{bt} + \Delta t_\mathrm{c}^\mathrm{act}$
Valves: aortic (AV), pulmonary (PV), mitral (MV), and tricuspid (TV) valve; pulmonary (PO) and systemic (SO) outlet			
A_v^open	500 (MV, TV) 400 (AV, PV, SO, PO)	mm^2	Valve cross-sectional area
A_v^leak	0 (AV, PV, MV, TV) A_v^open (SO, PO)	mm^2	Cross-sectional area of closed/regurging valve
Periphery: systemic (sys) and pulmonary (pulm) circulation			
$\Delta p_{py}^\mathrm{ref}$	1.5 (pulm) 10.0 (sys)	kPa	Blood pressure drop in pulmonary/systemic circulation
q_{py}^ref	85 (pulm, sys)	mL/s	Reference pulmonary/systemic flow
r_{py}	1 (pulm) 2 (sys)	[–]	Resistance scaling factor

where A_v^leak is the given valve cross-sectional areas of the closed (regurging) valve. Using this the current cross sectional area of the valve is

$$A_v = \begin{cases} A_v^\mathrm{open} & \text{for } \Delta p_v > 0, \\ A_v^\mathrm{leak} & \text{for } \Delta p_v < 0 \text{ and } q_v < 0, \\ A_v^\mathrm{closing} & \text{for } \Delta p_v < 0 \text{ and } q_v > 0. \end{cases} \tag{A.40}$$

We define

$$A_v^\mathrm{min} = \min\left(A_v^\mathrm{prox}, A_v, A_v^\mathrm{dist}\right), \tag{A.41}$$

with A_v^prox and A_v^dist the cross-sectional area of the proximal and distal cavities or tubes respectively, see (A.1), (A.18), (A.28) and Fig. A.10. Using this β_v is given as

$$\beta_v = \begin{cases} \frac{1}{2}\rho_\mathrm{b}\left[\left(\frac{1}{A_v^\mathrm{min}}\right)^2 - \left(\frac{1}{A_v^\mathrm{prox}}\right)^2\right] & \text{for } q_v \geq 0, \\ \frac{1}{2}\rho_\mathrm{b}\left[\left(\frac{1}{A_v^\mathrm{dist}}\right)^2 - \left(\frac{1}{A_v^\mathrm{min}}\right)^2\right] & \text{for } q_v < 0. \end{cases} \tag{A.42}$$

Flow over the valve is finally updated using (A.37) by

$$\dot{q}_v = \frac{\Delta p_v - \beta_v q_v^2}{\alpha_v}. \tag{A.43}$$

A.9. Solve ODE system

A Runge–Kutta–Fehlberg method (RKF45), see, e.g., [104], is used to solve the system of 26 ordinary differential equations (ODEs):

8 ODEs: for each of the four tubes and the four cavities we get an ODE to update the volume using Eqs. (A.33) and (A.34).

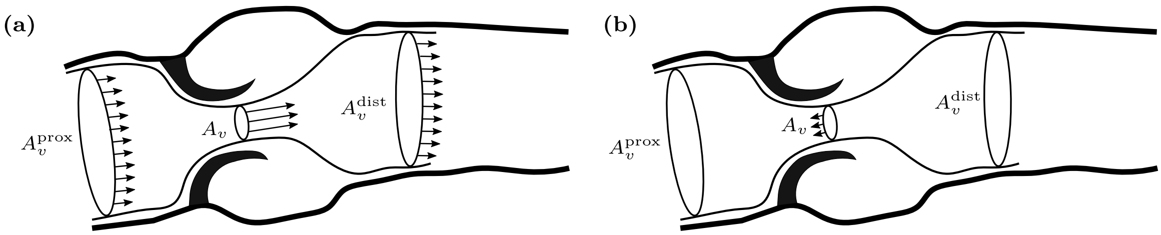

Fig. A.10. Schematic of the (a) open and (b) regurging valve.
Source: Based on [103].

Table A.5
Default parameters for the *CircAdapt* model, fitted to general experimental data in [44].

Parameter	Value	Unit	Description
Tubes: aorta (AO), arteria pulmonalis (AP), venae cavae (VC), and venae pulmonales (VP)			
p_t^{ref}	12.0 (AO) 0.5 (VP)<br>0.12 (VC) 1.8 (AP)	kPa	reference tube pressure
Sarcomeres in left (LV) and right (RV) ventricle; left (LA) and right atrium (RA)			
$L^{\text{s,ref}}$	2.00	µm	Reference sarcomere length
$L^{\text{elast,iso}}$	0.04	µm	Length of isometrically stressed series elastic element
$v^{\max}$	7 (LV, RV) 14 (LA, RA)	µm/s	Reference shortening velocity
$t_c^{\text{act,ref}}$	$0.5\,t_{\text{cycle}}$ (LV, RV) $0.15\,t_{\text{cycle}}$ (LA, RA)	s	Reference duration of contraction
τ^{R}	0.25 (LV, RV) 0.4 (LA, RA)	[–]	Ratio rise time to $t_c^{\text{act,ref}}$
τ^{D}	0.25 (LV, RV) 0.4 (LA, RA)	[–]	Ratio decay time to $t_c^{\text{act,ref}}$
$L^{\text{act0,ref}}$	1.51	µm	Contractile element length with zero active stress
$L^{\text{pas0,ref}}$	1.80	µm	Sarcomere length with zero passive stress
$\sigma^{\text{act,max}}$	120 (LV, RV) 84 (LA, RA)	kPa	Maximal isometric stress
$\sigma^{\text{pas,max}}$	22 (LV, RV) 50 (LA, RA)	kPa	Maximal passive stress
$\mathrm{d}L^{\text{s,pas}}$	0.6	µm	
TriSeg module			
τ_{Sep}	0.005	[–]	Time constant
Pericardium			
$p_{\text{peri}}^{\text{ref}}$	0.005	[–]	Constant reference pressure
$V_{\text{peri}}^{\text{ref}}$	0.005	[–]	Constant reference volume
Periphery: systemic (sys) and pulmonary (pulm) circulation			
k_{py}	2 (pulm) 1 (sys)	[–]	Nonlinearity exponent

2 ODEs: for the septum we update midwall volume and the radius according to (A.26).
10 ODEs: for the sarcomeres of each cavity and the septum we update sarcomere contracting length and contractility using (A.7)–(A.8).
6 ODEs: for each of the four valves and the two outlets we update flow by (A.43).

Appendix B. Finite element formulation

B.1. Variational formulation

We first ignore the acceleration term in (3) and look at the stationary version of the boundary value problem (3)–(4) and (15). For the full nonlinear elastodynamics problem see Appendix C. The stationary boundary value problem is formally equivalent to the equations

$$\langle \mathcal{A}_0(\mathbf{u}), \mathbf{v}\rangle_{\Omega_0} - \langle \mathcal{F}_0(\mathbf{u}, p_c), \mathbf{v}\rangle_{\Omega_0} = \mathbf{0}, \tag{B.1}$$

$$\langle V_c^{\text{PDE}}(\mathbf{u}), q\rangle_{\Omega_0} - \langle V_c^{\text{ODE}}(p_c), q\rangle_{\Omega_0} = 0, \tag{B.2}$$

which is valid for all smooth enough vector fields $\mathbf{v}$ vanishing on the Dirichlet boundary $\Gamma_{0,\mathrm{D}}$, testfunctions q that are 1 for the cavity c and 0 otherwise, the duality pairing $\langle\cdot,\cdot\rangle_{\Omega_0}$, and cavities $c \in \{\mathrm{LV}, \mathrm{RV}, \mathrm{LA}, \mathrm{RA}\}$. The second term on the left hand side of the variational equation (B.1) has the physical interpretation of the rate of internal mechanical work and is given by

$$\langle \mathcal{A}_0(\mathbf{u}), \mathbf{v}\rangle_{\Omega_0} := \int_{\Omega_0} \mathbf{S}(\mathbf{u}) : \boldsymbol{\Sigma}(\mathbf{u},\mathbf{v})\,\mathrm{d}\mathbf{X}, \tag{B.3}$$

with the second Piola–Kirchhoff stress tensor $\mathbf{S}$, see (5), and the directional derivative of the Green–Lagrange strain tensor $\boldsymbol{\Sigma}(\mathbf{u},\mathbf{v})$, see [41,105]. The weak form of the contribution of pressure loads (B.1), right term, is computed using (4)

$$\langle \mathcal{F}_0(\mathbf{u}, p_c), \mathbf{v}\rangle_{\Omega_0} = -p_c \int_{\Gamma_{0,\mathrm{N}}} J\,\mathbf{F}^{-\top}(\mathbf{u})\,\mathbf{n}_0^{\mathrm{out}} \cdot \mathbf{v}\;\mathrm{d}s_{\mathbf{X}}. \tag{B.4}$$

The first term of the coupling equation (B.2) is computed from (14) using Nanson's formula and $\mathbf{x} = \mathbf{X} + \mathbf{u}$ by

$$\langle V_c^{\mathrm{PDE}}(\mathbf{u}), q\rangle_{\Omega_0} = \frac{1}{3}\int_{\Gamma_{0,\mathrm{N}}} (\mathbf{X}+\mathbf{u}) \cdot J\mathbf{F}^{-\top}\mathbf{n}_0^{\mathrm{out}}\, q\;\mathrm{d}s_{\mathbf{X}}, \tag{B.5}$$

The second term of (B.2) is computed using the lumped *CircAdapt* model, see Section 2.4, for $c \in \{\mathrm{LV}, \mathrm{RV}, \mathrm{LA}, \mathrm{RA}\}$.

B.2. Consistent linearization

To solve the nonlinear variational equations (B.1)–(B.2), with a FE approach we first apply a Newton–Raphson scheme, see [77]. Given a nonlinear and continuously differentiable operator $F\colon X \to Y$ a solution to $F(x) = 0$ can be approximated by

$$\begin{aligned} x^{k+1} &= x^k + \Delta x,\\ \left.\frac{\partial F}{\partial x}\right|_{x=x^k} \Delta x &= -F(x^k), \end{aligned}$$

which is looped until convergence. In our case, we have $X = \left[H^1(\Omega_0, \Gamma_{0,\mathrm{D}})\right]^3 \times \mathbb{R}$, $Y = \mathbb{R}^2$, $\Delta x = (\Delta\mathbf{u}, \Delta p_c)^\top$, $x^k = (\mathbf{u}^k, p_c^k)^\top$, and $F = (R_{\mathbf{u}}, R_{\mathrm{p}})^\top$. We obtain the following linearized saddle-point problem for each $(\mathbf{u}^k, p_c^k) \in \left[H^1(\Omega_0, \Gamma_{0,\mathrm{D}})\right]^3 \times \mathbb{R}$, find $(\Delta\mathbf{u}, \Delta p_c) \in \left[H_0^1(\Omega_0)\right]^3 \times \mathbb{R}$ such that

$$\begin{aligned} &\langle \Delta\mathbf{u}, A_0'(\mathbf{u}^k)\,\mathbf{v}\rangle_{\Omega_0} + \langle \Delta\mathbf{u}, \mathcal{F}_0'(\mathbf{u}^k, p_c^k)\,\mathbf{v}\rangle_{\Omega_0}\\ &\qquad + \langle \Delta p_c, \mathcal{F}_0'(\mathbf{u}^k, p_c^k)\,\mathbf{v}\rangle_{\Omega_0} = -\langle R_{\mathbf{u}}(\mathbf{u}^k, p_c^k), \mathbf{v}\rangle_{\Omega_0}, && \text{(B.6)}\\ &\langle \Delta\mathbf{u}, V_c^{\mathrm{PDE}}(\mathbf{u}^k)\,q\rangle_{\Omega_0} - \langle \Delta p_c, V_c^{\mathrm{ODE}}(p_c^k)\,q\rangle_{\Omega_0} = -\langle R_{\mathrm{p}}(\mathbf{u}^k, p_c^k), q\rangle_{\Omega_0}, && \text{(B.7)} \end{aligned}$$

with the updates

$$\begin{aligned} \mathbf{u}^{k+1} &= \mathbf{u}^k + \Delta\mathbf{u}, && \text{(B.8)}\\ p_c^{k+1} &= p_c^k + \Delta p_c, && \text{(B.9)} \end{aligned}$$

and the particular terms are introduced below. The Gâteaux derivative of (B.1) with respect to the displacement change update $\Delta\mathbf{u}$ yields the first

$$\begin{aligned} \langle \Delta\mathbf{u}, A_0'(\mathbf{u}^k)\,\mathbf{v}\rangle_{\Omega_0} &:= D_{\Delta\mathbf{u}}\langle \mathcal{A}_0(\mathbf{u}), \mathbf{v}\rangle_{\Omega_0}\big|_{\mathbf{u}=\mathbf{u}^k}\\ &= \int_{\Omega_0} \mathbf{S}_k : \boldsymbol{\Sigma}(\Delta\mathbf{u}, \mathbf{v})\,\mathrm{d}\mathbf{X} + \int_{\Omega_0} \boldsymbol{\Sigma}(\mathbf{u}^k, \Delta\mathbf{u}) : \mathbb{C}_k : \boldsymbol{\Sigma}(\mathbf{u}^k, \mathbf{v})\,\mathrm{d}\mathbf{X}, \end{aligned} \tag{B.10}$$

and second term of (B.6)

$$\begin{aligned} \langle \Delta\mathbf{u}, \mathcal{F}_0'(\mathbf{u}^k, p_c^k)\,\mathbf{v}\rangle_{\Omega_0} &:= D_{\Delta\mathbf{u}}\langle \mathcal{F}_0(\mathbf{u}, p_c), \mathbf{v}\rangle_{\Omega_0}\big|_{\mathbf{u}=\mathbf{u}^k, p_c=p_c^k}\\ &= p_c^k \int_{\Gamma_{0,\mathrm{N}}} J_k\mathbf{F}_k^{-\top}\,\mathrm{Grad}^\top\Delta\mathbf{u}\,\mathbf{F}_k^{-\top}\mathbf{n}_0^{\mathrm{out}} \cdot \mathbf{v}\;\mathrm{d}s_{\mathbf{X}}\\ &\quad - p_c^k \int_{\Gamma_{0,\mathrm{N}}} J_k(\mathbf{F}_k^{-\top} : \mathrm{Grad}\,\Delta\mathbf{u})\,\mathbf{F}_k^{-\top}\mathbf{n}_0^{\mathrm{out}} \cdot \mathbf{v}\;\mathrm{d}s_{\mathbf{X}}, \end{aligned} \tag{B.11}$$

with abbreviations

$$\mathbf{F}_k := \mathbf{F}(\mathbf{u}^k),\ \ J_k := \det(\mathbf{F}^k),\ \ \mathbf{S}_k := \mathbf{S}|_{\mathbf{u}=\mathbf{u}^k}\ ,\ \ \mathbb{C}_k := \mathbb{C}|_{\mathbf{u}=\mathbf{u}^k}\ .$$

The Gâteaux derivative of (B.1) with respect to the pressure change update Δp_c yields the third term of (B.6)

$$\begin{aligned}\langle \Delta p_c, \mathcal{F}_0'(\mathbf{u}^k, p_c^k)\,\mathbf{v}\rangle_{\Omega_0} &:= D_{\Delta p_c}\langle \mathcal{F}_0(\mathbf{u}, p_c), \mathbf{v}\rangle_{\Omega_0}\big|_{\mathbf{u}=\mathbf{u}^k, p_c=p_c^k}\\ &= -\Delta p_c \int_{\Gamma_{0,\mathrm{N}}} J_k\,\mathbf{F}_k^{-\top}\,\mathbf{n}_0^{\mathrm{out}}\cdot\mathbf{v}\ \mathrm{d}s_{\mathbf{X}}.\end{aligned} \tag{B.12}$$

The residual $R_{\mathbf{u}}$, i.e., the right hand side of (B.6), is computed as

$$\langle R_{\mathbf{u}}(\mathbf{u}^k, p_c^k), \mathbf{v}\rangle_{\Omega_0} := \langle A_0(\mathbf{u}^k), \mathbf{v}\rangle_{\Omega_0} - \langle \mathcal{F}_0(\mathbf{u}^k, p_c^k), \mathbf{v}\rangle_{\Omega_0}. \tag{B.13}$$

From (B.5), using the known relations, see, e.g., [105],

$$\frac{\partial J}{\partial \mathbf{F}} : \mathrm{Grad}\,\Delta\mathbf{u} = J\mathbf{F}^{-\top} : \mathrm{Grad}\,\Delta\mathbf{u}$$

$$\frac{\partial \mathbf{F}^{-\top}}{\partial \mathbf{F}} : \mathrm{Grad}\,\Delta\mathbf{u} = -\mathbf{F}^{-\top}(\mathrm{Grad}\,\Delta\mathbf{u})^{\top}\mathbf{F}^{-\top}$$

we can calculate the first term of (B.7) as the Gâteaux derivative with respect to the update $\Delta\mathbf{u}$

$$\begin{aligned}\langle \Delta\mathbf{u}, V_c^{\mathrm{PDE}}(\mathbf{u}^k)\,q\rangle_{\Omega_0} &:= D_{\Delta\mathbf{u}}\langle V_c^{\mathrm{PDE}}(\mathbf{u}), q\rangle_{\Omega_0}\big|_{\mathbf{u}=\mathbf{u}^k}\\ &= D_{\Delta\mathbf{u}}\frac{1}{3}\int_{\Gamma_{0,\mathrm{N}}}\left(\mathbf{X}+\mathbf{u}^k\right)\cdot J_k\mathbf{F}_k^{-\top}\mathbf{n}_0^{\mathrm{out}}\,q\ \mathrm{d}s_{\mathbf{X}}\\ &= \frac{1}{3}\int_{\Gamma_{0,\mathrm{N}}} J_k(\mathbf{F}_k^{-\top} : \mathrm{Grad}\,\Delta\mathbf{u})\mathbf{x}\cdot\mathbf{F}_k^{-\top}\mathbf{n}_0^{\mathrm{out}}\,q\ \mathrm{d}s_{\mathbf{X}}\\ &\quad -\frac{1}{3}\int_{\Gamma_{0,\mathrm{N}}} J_k\mathbf{x}\cdot\mathbf{F}_k^{-\top}(\mathrm{Grad}\,\Delta\mathbf{u})^{\top}\mathbf{F}_k^{-\top}\mathbf{n}_0^{\mathrm{out}}\,q\ \mathrm{d}s_{\mathbf{X}}\\ &\quad +\frac{1}{3}\int_{\Gamma_{0,\mathrm{N}}} J_k\Delta\mathbf{u}\cdot\mathbf{F}_k^{-\top}\mathbf{n}_0^{\mathrm{out}}\,q\ \mathrm{d}s_{\mathbf{X}},\end{aligned} \tag{B.14}$$

with q a testfunction that is 1 for the surface of cavity c, $\Gamma_{0,c}$, and 0 otherwise.

The second term of (B.7) is computed as a numerical derivative

$$\begin{aligned}\langle \Delta p_c, V_c^{\mathrm{ODE}}(p_c^k)\,q\rangle_{\Omega_0} &:= D_{\Delta p_c}\langle V_c^{\mathrm{ODE}}(p_c)\,q\rangle_{\Omega_0}\big|_{p_c=p_c^k}\\ &= \frac{1}{\epsilon}\left(V_c^{\mathrm{ODE}}(p_c^k+\epsilon) - V_c^{\mathrm{ODE}}(p_c^k)\right)q,\end{aligned} \tag{B.15}$$

where $\epsilon = p_c^k\sqrt{\epsilon_{\mathrm{m}}}$ is chosen according to [106, Chapter 5.7] with $\epsilon_{\mathrm{m}} = 2^{-52} \approx 2.2 * 10^{-16}$ the machine accuracy.

Finally, the residual R_{p}, i.e., the right hand side of (B.7), is computed as

$$\langle R_{\mathrm{p}}(\mathbf{u}^k, p_c^k), q\rangle_{\Omega_0} := \langle V_c^{\mathrm{PDE}}(\mathbf{u}), q\rangle_{\Omega_0} - \langle V_c^{\mathrm{ODE}}(p_c), q\rangle_{\Omega_0}. \tag{B.16}$$

B.3. Assembling of the block matrices

To apply the finite element method (FEM) we consider an admissible decomposition of the computational domain $\Omega \subset \mathbb{R}^3$ into M tetrahedral elements τ_j and introduce a conformal finite element space

$$X_h \subset H^1(\Omega_0),\ \ N = \dim X_h$$

of piecewise polynomial continuous basis functions φ_i. The linearized variational problem (B.6)–(B.7) and a Galerkin FE discretization result in solving the block system to find $\delta\underline{u} \in \mathbb{R}^{3N}$ and $\delta\underline{p}_{\mathcal{C}} \in \mathbb{R}^{N_{\mathrm{cav}}}$ such that

$$\mathbf{K}'(\underline{u}^k, \underline{p}_{\mathcal{C}}^k)\begin{pmatrix}\delta\underline{u}\\ \delta\underline{p}_{\mathcal{C}}\end{pmatrix} = -\underline{K}(\underline{u}^k, \underline{p}_{\mathcal{C}}^k),\quad \underline{K}(\underline{u}^k, \underline{p}_{\mathcal{C}}^k) := -\begin{pmatrix}\underline{R}_{\mathbf{u}}(\underline{u}^k, \underline{p}_{\mathcal{C}}^k)\\ \underline{R}_p(\underline{u}^k, \underline{p}_{\mathcal{C}}^k)\end{pmatrix},$$

i.e.,

$$\begin{pmatrix} (\mathbf{A}'-\mathbf{M}')(\underline{u}^k,\underline{p}_{\mathcal{C}}^k) & \mathbf{B}_{\mathrm{p}}'(\underline{u}^k) \\ \mathbf{B}_{\mathbf{u}}'(\underline{u}^k) & \mathbf{C}'(\underline{p}_{\mathcal{C}}^k) \end{pmatrix} \begin{pmatrix} \delta\underline{u} \\ \delta\underline{p}_{\mathcal{C}} \end{pmatrix} = -\begin{pmatrix} \underline{A}(\underline{u}^k) - \underline{B}_{\mathrm{p}}(\underline{u}^k,\underline{p}_{\mathcal{C}}^k) \\ \underline{V}_c^{\mathrm{PDE}}(\underline{u}^k) - \underline{V}_c^{\mathrm{ODE}}(\underline{p}_{\mathcal{C}}^k) \end{pmatrix}, \tag{B.17}$$

$$\underline{u}^{k+1} = \underline{u}^k + \delta\underline{u}, \tag{B.18}$$

$$\underline{p}_{\mathcal{C}}^{k+1} = \underline{p}_{\mathcal{C}}^k + \delta\underline{p}_{\mathcal{C}} \tag{B.19}$$

with the solution vectors $\underline{u}^k \in \mathbb{R}^{3N}$ and $\underline{p}_{\mathcal{C}}^k \in \mathbb{R}^{N_{\mathrm{cav}}}$ at the kth Newton step. The tangent stiffness matrix $\mathbf{A}' \in \mathbb{R}^{3N\times 3N}$ is calculated from (B.10) according to

$$\mathbf{A}'(\underline{u}^k)[j,i] := \langle \boldsymbol{\varphi}_i, \mathcal{A}_0'(\mathbf{u}^k)\,\boldsymbol{\varphi}_j\rangle_{\Omega_0} \tag{B.20}$$

and the mass matrix $\mathbf{M}' \in \mathbb{R}^{3N\times 3N}$ is calculated from (B.11) according to

$$\mathbf{M}'(\underline{u}^k,\underline{p}_{\mathcal{C}}^k)[j,i] := \langle \boldsymbol{\varphi}_i, \mathcal{F}_0'(\mathbf{u}^k,p_c^k)\,\boldsymbol{\varphi}_j\rangle_{\Omega_0}, \tag{B.21}$$

see also [41,105].

The off-diagonal matrices $\mathbf{B}_{\mathbf{u}}' \in \mathbb{R}^{3N\times N_{\mathrm{cav}}}$ and $\mathbf{B}_{\mathrm{p}}' \in \mathbb{R}^{N_{\mathrm{cav}}\times 3N}$ in (B.17) are assembled using (B.14)

$$\mathbf{B}_{\mathbf{u}}'(\underline{u}^k,\underline{p}_{\mathcal{C}}^k)[i,j] = \langle \boldsymbol{\varphi}_j, V_c^{\mathrm{PDE}}(\mathbf{u}^k)\hat{\varphi}_i\rangle_{\Omega_0}, \quad i=1,\dots,N_{\mathrm{cav}} \tag{B.22}$$

and using (B.12)

$$\mathbf{B}_{\mathrm{p}}'(\underline{u}^k,\underline{p}_{\mathcal{C}}^k)[i,j] = \langle \hat{\varphi}_j, \mathcal{F}_0'(\mathbf{u}^k,p_c^k)\boldsymbol{\varphi}_i\rangle_{\Omega_0}, \quad j=1,\dots,N_{\mathrm{cav}}, \tag{B.23}$$

with the constant shape function $\hat{\varphi}_j = 1$ if $\tau_j \in \Gamma_{0,c}$ and $\hat{\varphi}_j = 0$ if $\tau_j \notin \Gamma_{0,c}$ for $c \in \{\mathrm{LV},\mathrm{RV},\mathrm{LA},\mathrm{RA}\}$.

Using a technique as described in [64, Sect. 4.2] this assembling procedure can be simplified for closed cavities such that

$$\mathbf{B}_{\mathrm{p}}'(\underline{u}^k,\underline{p}_{\mathcal{C}}^k) = \left[\mathbf{B}_{\mathbf{u}}'(\underline{u}^k,\underline{p}_{\mathcal{C}}^k)\right]^\top.$$

The circulatory compliance matrix $\mathbf{C}'(\underline{p}_{\mathcal{C}}^k) \in \mathbb{R}^{N_{\mathrm{cav}}\times N_{\mathrm{cav}}}$ is computed from (B.15) as

$$\mathbf{C}'(\underline{p}_{\mathcal{C}}^k)[i,j] = \langle \hat{\varphi}_j, V_c^{\mathrm{ODE}}(p_c^k)\,\hat{\varphi}_i\rangle_{\Omega_0}, \quad i,j=1,\dots,N_{\mathrm{cav}}, \tag{B.24}$$

with the constant shape function $\hat{\varphi}_i, \hat{\varphi}_j = 1$ for cavity c and 0 otherwise, leading to a diagonal matrix.

The terms on the upper right hand side $\underline{A} \in \mathbb{R}^{3N}$, $\underline{B}_{\mathrm{p}} \in \mathbb{R}^{3N}$ are constructed using (B.13) resulting in $\underline{R}_{\mathbf{u}}(\underline{u}^k,\underline{p}_{\mathcal{C}}^k) = \underline{A}(\underline{u}^k) - \underline{B}_{\mathrm{p}}(\underline{u}^k,\underline{p}_{\mathcal{C}}^k)$ with

$$\underline{A}(\underline{u}^k)[i] := \langle \mathcal{A}_0(\mathbf{u}^k), \boldsymbol{\varphi}_i\rangle_{\Omega_0} \tag{B.25}$$

and

$$\underline{B}_{\mathrm{p}}(\underline{u}^k,\underline{p}_{\mathcal{C}}^k)[i] := \langle \mathcal{F}_0(\mathbf{u}^k,p_c^k), \boldsymbol{\varphi}_i\rangle_{\Omega_0}. \tag{B.26}$$

Finally, the lower right hand side in (B.17), $\underline{R}_p(\underline{u}^k,\underline{p}_c^k) = \underline{V}^{\mathrm{PDE}}(\underline{u}^k) - \underline{V}^{\mathrm{ODE}}(\underline{p}_c^k) \in \mathbb{R}^{N_{\mathrm{cav}}}$, is assembled from (B.16) with

$$\underline{V}^{\mathrm{PDE}}(\underline{u}^k)[i] = \langle V_c^{\mathrm{PDE}}(\mathbf{u}), \hat{\varphi}_i\rangle_{\Omega_0}, \quad i=1,\dots,N_{\mathrm{cav}}, \tag{B.27}$$

and

$$\underline{V}^{\mathrm{ODE}}(\underline{p}_c^k)[i] = \langle V_c^{\mathrm{ODE}}(p_c), \hat{\varphi}_i\rangle_{\Omega_0}, \quad i=1,\dots,N_{\mathrm{cav}}. \tag{B.28}$$

Appendix C. Generalized-α scheme

After standard discretization we rewrite Eq. (3) using Eqs. (B.25) and (B.26) as a nonlinear ODE reading

$$\rho_0\mathbf{M}_\alpha\ddot{\underline{u}}(t) + \underline{R}_{\mathbf{u}}(\underline{u},t) = \underline{0}, \tag{C.1}$$

with the mass matrix

$$\mathbf{M}_\alpha[i,j] := \int_{\Omega_0} \boldsymbol{\varphi}_i(\mathbf{X})\cdot\boldsymbol{\varphi}_j(\mathbf{X})\,\mathrm{d}\mathbf{X}.$$

Following [107] we reformulate Eq. (C.1) as a first order ODE system by introducing the velocity $\underline{v}$

$$\rho_0 \mathbf{M}_\alpha \dot{\underline{v}}(t) + \underline{R}_{\mathbf{u}}(\underline{u}, t) = \underline{0}, \tag{C.2}$$

$$\mathbf{M}_\alpha \dot{\underline{u}}(t) - \mathbf{M}_\alpha \underline{v}(t) = \underline{0} \tag{C.3}$$

and apply a generalized-α approach [108]. To this end we define three parameters

$$\alpha_\mathrm{f} := \frac{1}{1+\rho_\infty}, \quad \alpha_\mathrm{m} := \frac{3-\rho_\infty}{2(1+\rho_\infty)}, \quad \gamma := \frac{1}{2} + \alpha_\mathrm{m} - \alpha_\mathrm{f},$$

where the *spectral radius* ρ_∞ is a parameter between 0 and 1. With this we introduce

$$\begin{aligned}
\dot{\underline{v}}_{n+\alpha_\mathrm{m}} &:= \alpha_\mathrm{m} \dot{\underline{v}}_{n+1} + (1-\alpha_\mathrm{m}) \dot{\underline{v}}_n, \\
\dot{\underline{u}}_{n+\alpha_\mathrm{m}} &:= \alpha_\mathrm{m} \dot{\underline{u}}_{n+1} + (1-\alpha_\mathrm{m}) \dot{\underline{u}}_n, \\
\underline{v}_{n+\alpha_\mathrm{f}} &:= \alpha_\mathrm{f}\, \underline{v}_{n+1} + (1-\alpha_\mathrm{f})\, \underline{v}_n, \\
\underline{u}_{n+\alpha_\mathrm{f}} &:= \alpha_\mathrm{f}\, \underline{u}_{n+1} + (1-\alpha_\mathrm{f})\, \underline{u}_n,
\end{aligned}$$

and reformulate Eq. (C.2) as

$$\rho_0 \mathbf{M}_\alpha \dot{\underline{v}}_{n+\alpha_\mathrm{m}} + \underline{R}_{\mathbf{u}}(\underline{u}_{n+\alpha_\mathrm{f}}) = \underline{0}, \tag{C.4}$$

$$\mathbf{M}_\alpha \dot{\underline{u}}_{n+\alpha_\mathrm{m}} - \mathbf{M}_\alpha \underline{v}_{n+\alpha_\mathrm{f}} = \underline{0}. \tag{C.5}$$

Here, the second equation gives us

$$\dot{\underline{u}}_{n+\alpha_\mathrm{m}} = \underline{v}_{n+\alpha_\mathrm{f}}$$

and we get for the velocity update

$$\underline{v}_{n+1} = \frac{\alpha_\mathrm{m}}{\alpha_\mathrm{f}\gamma \Delta t}\left(\underline{u}_{n+1} - \underline{u}_n\right) + \frac{\gamma - \alpha_\mathrm{m}}{\gamma \alpha_\mathrm{f}} \dot{\underline{u}}_n + \frac{\alpha_\mathrm{f} - 1}{\alpha_\mathrm{f}} \underline{v}_n. \tag{C.6}$$

From this and the relationship by Newmark [109]

$$\begin{aligned}
\underline{u}_{n+1} &= \underline{u}_n + \Delta t \left(\gamma \dot{\underline{u}}_{n+1} + (1-\gamma)\dot{\underline{u}}_n\right), \\
\underline{v}_{n+1} &= \underline{v}_n + \Delta t \left(\gamma \dot{\underline{v}}_{n+1} + (1-\gamma)\dot{\underline{v}}_n\right),
\end{aligned}$$

we obtain

$$\dot{\underline{v}}_{n+1} = \frac{\alpha_\mathrm{m}}{\alpha_\mathrm{f}\gamma^2 \Delta t^2}\left(\underline{u}_{n+1} - \underline{u}_n\right) - \frac{1}{\alpha_\mathrm{f}\gamma \Delta t} \underline{v}_n + \frac{\gamma - 1}{\gamma} \dot{\underline{v}}_n + \frac{\gamma - \alpha_\mathrm{m}}{\alpha_\mathrm{f}\gamma^2 \Delta t}. \tag{C.7}$$

Hence, we can rewrite the whole first order system only dependent on the unknowns $\underline{u}_{n+1}$.

Newton's method for the generalized-α scheme. For the implementation of Newton's method we compute

$$\frac{\partial \dot{\underline{v}}_{n+\alpha_\mathrm{m}}}{\partial \underline{u}_{n+1}} = \frac{\alpha_\mathrm{m}^2}{\alpha_\mathrm{f}\gamma^2 \Delta t^2}, \quad \frac{\partial \underline{v}_{n+\alpha_\mathrm{f}}}{\partial \underline{u}_{n+1}} = \frac{\alpha_\mathrm{f}\alpha_\mathrm{m}}{\alpha_\mathrm{f}\gamma \Delta t} = \frac{\alpha_\mathrm{m}}{\gamma \Delta t}, \quad \frac{\partial \underline{u}_{n+\alpha_\mathrm{f}}}{\partial \underline{u}_{n+1}} = \alpha_\mathrm{f}. \tag{C.8}$$

To calculate the solution at the current timestep we assume that we know $\underline{u}_n$, $\dot{\underline{u}}_n$, $\underline{v}_n$ and $\dot{\underline{v}}_n$ from the previous time step n and get from Eq. (C.4) for the residual

$$\underline{R}_\alpha(\underline{u}_{n+1}^k) := -\rho_0 \mathbf{M}_\alpha \dot{\underline{v}}_{n+\alpha_\mathrm{m}}^k - \underline{R}_{\mathbf{u}}(\underline{u}_{n+\alpha_\mathrm{f}}^k), \tag{C.9}$$

with $\dot{\underline{v}}_{n+\alpha_\mathrm{m}}^k := \dot{\underline{v}}_{n+\alpha_\mathrm{m}}(\underline{u}_{n+1}^k)$ and $\underline{u}_{n+\alpha_\mathrm{f}}^k := \underline{u}_{n+\alpha_\mathrm{f}}(\underline{u}_{n+1}^k)$. To increase stability we consider Rayleigh damping by adding the two matrices

$$\mathbf{D}_\mathrm{mass}(\underline{u}_{n+1}^k) = \rho_0 \quad \beta_\mathrm{mass} \mathbf{M}_\alpha \quad \underline{v}_{n+\alpha_\mathrm{f}}^k, \tag{C.10}$$

$$\mathbf{D}_\mathrm{stiff}(\underline{u}_{n+1}^k) = \beta_\mathrm{stiff}\, \mathbf{A}'(u_n) \underline{v}_{n+\alpha_\mathrm{f}}^k, \tag{C.11}$$

to the residual (C.9) with $\underline{v}_{n+\alpha_\mathrm{f}}^k := \underline{v}_{n+\alpha_\mathrm{f}}(\underline{u}_{n+1}^k)$ and Rayleigh damping parameters $\beta_\mathrm{mass} \geq 0\,\mathrm{ms}^{-1}$, $\beta_\mathrm{stiff} \geq 0\,\mathrm{ms}$.

The tangent stiffness matrix is now calculated using (C.8) as

$$\mathbf{A}'_{\alpha}(\underline{u}^k_{n+1}, \underline{p}^k_{\mathcal{C},n+1}) := \rho_0 \frac{\partial \underline{\dot{v}}_{n+\alpha_\mathrm{m}}}{\partial \underline{u}_{n+1}} \mathbf{M}_\alpha + \frac{\partial \underline{u}_{n+\alpha_\mathrm{f}}}{\partial \underline{u}_{n+1}} \left(\mathbf{A}'(\underline{u}^k_{n+\alpha_\mathrm{f}}) - \mathbf{M}'(\underline{u}^k_{n+\alpha_\mathrm{f}}, \underline{p}^k_{\mathcal{C},n+1}) \right)$$
$$= \rho_0 \frac{\alpha_\mathrm{m}^2}{\alpha_\mathrm{f} \gamma^2 \Delta t^2} \mathbf{M}_\alpha + \alpha_\mathrm{f} \left(\mathbf{A}'(\underline{u}^k_{n+\alpha_\mathrm{f}}) - \mathbf{M}'(\underline{u}^k_{n+\alpha_\mathrm{f}}, \underline{p}^k_{\mathcal{C},n+1}) \right), \quad \text{(C.12)}$$

with $\mathbf{A}'$, and $\mathbf{M}'$ being the known tangent stiffness matrices from the quasi-stationary elasticity case, see Eqs. (B.20) and (B.21). When using a coupling with the circulatory system we compute the off diagonal matrices and lower right hand side, see Eqs. (B.22), (B.23) and (B.27), in terms of $\underline{u}^k_{n+\alpha_\mathrm{f}}$.

Appendix D. Direct Schur complement solver for a small number of constraints

Given the block system $\mathbf{A} \in \mathbb{R}^{n\times n}$, $\mathbf{D} \in \mathbb{R}^{m\times m}$

$$\begin{pmatrix} \mathbf{A} & \mathbf{B} \\ \mathbf{C} & \mathbf{D} \end{pmatrix} \begin{pmatrix} \underline{x} \\ \underline{y} \end{pmatrix} = - \begin{pmatrix} \underline{f} \\ \underline{g} \end{pmatrix}$$

with

$$\mathbf{B} = \left(\underline{b}_1 \;\; | \;\; \cdots \;\; | \;\; \underline{b}_m\right) \in \mathbb{R}^{n\times m}, \quad \mathbf{C} = \left(\underline{c}_1 \;\; | \;\; \cdots \;\; | \;\; \underline{c}_m\right)^\top \in \mathbb{R}^{m\times n},$$

we can write the Schur complement system as

$$(\mathbf{C}\mathbf{A}^{-1}\mathbf{B} - \mathbf{D})\underline{y} = \underline{g} - \mathbf{C}\mathbf{A}^{-1}\underline{f}$$
$$\underline{x} = \mathbf{A}^{-1}\underline{f} - \mathbf{A}^{-1}\mathbf{B}\underline{y}.$$

With

$$\underline{r} = \mathbf{A}^{-1}\underline{f}, \quad \mathbf{S} = \mathbf{A}^{-1}\mathbf{B} = \left(\underline{s}_1 \;\; | \;\; \cdots \;\; | \;\; \underline{s}_m\right) \in \mathbb{R}^{n\times m}, \;\; \underline{s}_i = \mathbf{A}^{-1}\underline{b}_i, \;\; i = 1, \ldots, m \quad \text{(D.1)}$$

we get

$$(\mathbf{C}\mathbf{S} - \mathbf{D})\underline{y} = \underline{g} - \mathbf{C}\underline{r}$$
$$\underline{x} = \underline{r} - \mathbf{S}\underline{y}. \quad \text{(D.2)}$$

The realization of (D.2) involves $m + 1$ solves and the inversion of an $m \times m$ matrix. Since m is generally small this can be done symbolically.

$$[\mathbf{C}\mathbf{S}]_{ij} = \underline{c}_i \cdot \underline{s}_j, \;\; \text{for } i, j = 1, \ldots, m.$$

References

[1] L.J. Laslett, P. Alagona, B.A. Clark, J.P. Drozda, F. Saldivar, S.R. Wilson, C. Poe, M. Hart, The worldwide environment of cardiovascular disease: Prevalence, diagnosis, therapy, and policy issues: A report from the American College of Cardiology, J. Am. Coll. Cardiol. 60 (25 Supplement) (2012) S1–S49.

[2] A. Timmis, N. Townsend, C.P. Gale, A. Torbica, M. Lettino, S.E. Petersen, E.A. Mossialos, A.P. Maggioni, D. Kazakiewicz, H.T. May, et al., European society of cardiology: Cardiovascular disease statistics 2019, Eur. Heart J. 41 (1) (2020) 12–85.

[3] E. Wilkins, L. Wilson, K. Wickramasinghe, P. Bhatnagar, J. Leal, R. Luengo-Fernandez, R. Burns, M. Rayner, N. Townsend, European Cardiovascular Disease Statistics 2017, European Heart Network, 2017.

[4] N.P. Smith, A. de Vecchi, M. McCormick, D.A. Nordsletten, O. Camara, a.F. Frangi, H. Delingette, M. Sermesant, J. Relan, N. Ayache, M.W. Krueger, W.H.W. Schulze, R. Hose, I. Valverde, P. Beerbaum, C. Staicu, M. Siebes, J. Spaan, P.J. Hunter, J. Weese, H. Lehmann, D. Chapelle, R. Rezavi, euHeart: Personalized and integrated cardiac care using patient-specific cardiovascular modelling, Interface Focus 1 (2011) 349–364.

[5] H.J. Arevalo, F. Vadakkumpadan, E. Guallar, A. Jebb, P. Malamas, K.C. Wu, N.A. Trayanova, Arrhythmia risk stratification of patients after myocardial infarction using personalized heart models, Nature Commun. (2016).

[6] A. Prakosa, H.J. Arevalo, D. Deng, P.M. Boyle, P.P. Nikolov, H. Ashikaga, J.J.E. Blauer, E. Ghafoori, C.J. Park, R.C. Blake, F.T. Han, R.S. MacLeod, H.R. Halperin, D.J. Callans, R. Ranjan, J. Chrispin, S. Nazarian, N.A. Trayanova, Personalized virtual-heart technology for guiding the ablation of infarct-related ventricular tachycardia, Nature Biomed. Eng. (2018).

[7] M. Strocchi, M.A.F. Gsell, C.M. Augustin, O. Razeghi, C.H. Roney, A.J. Prassl, E.J. Vigmond, J.M. Behar, J.S. Gould, C.A. Rinaldi, M.J. Bishop, G. Plank, S.A. Niederer, Simulating ventricular systolic motion in a four-chamber heart model with spatially varying robin boundary conditions to model the effect of the pericardium, J. Biomech. 101 (2020) 109645.
